# Bacterial diversity in gut compartments of *Tomicus brevipilosus*: from shoot-boring preconditioning of *Pinus yunnanensis* to trunk-boring reproduction

**DOI:** 10.3389/fmicb.2026.1914356

**Published:** 2026-10-02

**Authors:** Mei-Ru Yang, Hua Xie, Shun-Yan Dong, Zong-Bo Li, Wei-Nan Kang

**Affiliations:** 1 Key Laboratory of Forest Disaster Warning and Control of Yunnan Province, School of Forestry, Southwest Forestry University, Kunming, China; 2 Yunxian Forestry and Grassland Bureau, Lincang, Yunnan, China

**Keywords:** gut bacterial communities, intestinal compartments, niche shift, SCFA-producing bacteria, *Tomicus brevipilosus*

## Abstract

**Background:**

Gut bacteria are critically important to bark beetles, especially under environmental fluctuations. *Tomicus brevipilosus* undergoes a niche shift from shoot-boring (which weakens *Pinus yunnanensis*) to trunk-boring for reproduction. The relationship between this transition and the gut microbiota remains to be elucidated.

**Methods:**

Using 16S rRNA sequencing, we characterized gut bacterial communities across adult feeding phase, sexes, and intestinal compartments.

**Results:**

The alimentary canal was composed of six morphologically distinguishable regions, to which Malpighian tubules were affixed. At the shoot-boring stage, no clear compartmentalization was observed in either sex, and no particular genus emerged as prominently associated. In trunk-boring adults, females undertake oviposition, whereas males evacuate excreta and food residues from the burrow entrance using their abdominal apex and elytral declivity. During the trunk-boring phase, compartment-specific differentiation was evident: the male rectum exhibited the highest bacterial ASV richness and complexity, harboring the greatest number of unique bacterial taxa at all taxonomic levels (phylum to genus), with significant enrichment of two short-chain fatty acid (SCFA)-producing genera (*Dietzia* and *Acinetobacter*), suggesting a potential defensive role against gallery pathogens. In contrast, female trunk-boring adults showed no clear gut compartmentalization, although the genera *Delftia* and *Halomonas* may contribute to cellulose breakdown and thereby facilitate reproduction. These functional inferences, however, await direct experimental confirmation. Interaction analyses further revealed that gut bacterial communities across intestinal segments varied significantly with both stage and sex.

**Conclusion:**

These stage- and sex-specific patterns likely reflect differing physiological and behavioral demands during the niche shift. Our findings demonstrate that this transition drives structural alterations in gut bacterial communities across both compartments and sexes, and provide a basis for future exploration of symbiont-based management strategies for this destructive forest pest.

## 1 Introduction

Bark beetles constitute an escalating global threat and are now widely recognized as a significant disturbance agent in forest ecosystems (Biedermann et al., 2019). The metabolic capabilities of bacteria can benefit bark beetles in several ways: providing certain nutrients (e.g., nitrogen) from nutritionally limited host tissues (Douglas, 2009), inhibiting antagonistic microorganisms (Therrien et al., 2015), degrading cellulose (Morales-Jiménez et al., 2012), and producing pheromones (Koski et al., 2024). Moreover, previous studies have established that gut-associated bacterial communities in bark beetles exhibit pronounced dynamics in response to environmental and physiological shifts, including dietary transitions (e.g., in *Ips typographus*) (Peral-Aranega et al., 2023), different life stages (e.g., in *I. typographus*, *I. sexdentatus* and *I. acuminatus*) (Chakraborty et al., 2023; Khara et al., 2024), and sex differentiation (e.g., in *Dendroctonus armandi*) (Hu et al., 2013). Given this, *T. brevipilosus* adults are of particular interest, as they undergo a distinct ecological niche shift from shoot-feeding to trunk-boring. These two feeding phases differ substantially in diet, season, reproductive status, behavior, and plant tissue type. We therefore propose that the functionally distinct gut bacteria likely act as key facilitators, rather than merely as general beneficial associates, directly enabling *T. brevipilosus* to surmount stage-specific pressures and accomplish its niche shift.

On a global scale, *Tomicus brevipilosus* has been documented to infest *Pinus koraiensis*, *P. insularis*, and *P. sylvestris* (Lieutier et al., 2015). Within Yunnan Province, *P. yunnanensis* serves as the dominant host for *T. brevipilosus* (Lu et al., 2014). Newly emerged *Tomicus brevipilosus* adults exit the host pines in which they developed and typically begin flying to the crowns of nearby trees, where they feed inside shoots until reaching sexual maturity (Chen et al., 2015). During this shoot-boring stage, intensive feeding on shoots by the beetles can lead to growth loss and reduced natural resistance of the trees (Chen et al., 2015). Sexually mature beetles then shift from shoots to trunks, initiating the trunk-boring (reproductive) stage. This transition from the shoot-boring stage to the trunk-boring stage represents a critical ecological shift that may drive a concomitant reorganization of the gut bacterial community. During trunk-boring stage, after females bore into the host and release aggregation pheromones to attract mates, males perform a brief courtship (including acoustic signals) that precedes mating; subsequently, females oviposit while males clear gallery debris (Ye and Ding, 1999; Långström et al., 2002; Lieutier et al., 2003; Chen et al., 2015; Wang et al., 2015). At this stage, the beetles interrupt vascular flow, rapidly triggering host tree death (Långström, 1983; Ye and Ding, 1999; Långström et al., 2002; Fernández et al., 2010). However, despite this well-characterized behavioral sequence, how the specific male cleaning and female oviposition behaviors directly drive adaptive changes in gut microbiota-or whether such changes are merely coincidental-has not been empirically addressed. Consequently, the functional significance of gut bacteria during this niche transition remains a major gap in the current literature.

Previous studies have demonstrated that gut bacteria confer diverse metabolic capabilities on bark beetles. For *T. brevipilosus*, the shoot-boring and trunk-boring stages differ markedly in diet, season, reproductive status, behavior, and plant tissue type. Such a niche shift is likely to impose selective pressures on the gut bacterial community, thereby driving adaptive reorganization. Consequently, elucidating how bacterial community structure responds to this ecological transition is critical for understanding the adaptive role of the microbiome in the life-history strategy of this beetle. On the basis of these considerations, we propose the following three hypotheses: (1) adult *T. brevipilosus* harbor stage-specific gut bacterial communities during the shoot-boring and trunk-boring stages, enabling them to cope with distinct survival conditions; (2) sex-specific differences exist in gut bacterial communities between the two stages; and (3) the bacterial communities inhabiting different gut compartments exhibit compositional differentiation across life stages and between sexes.

## 2 Methods

### 2.1 Insect rearing and sampling

*Tomicus brevipilosus* adults were collected alive from naturally infested *Pinus yunnanensis* trees in Malong District, Qujing City, Yunnan Province. Two distinct life-history stages were targeted: the shoot-boring stage (November), during which adults feed continuously on non-lignified shoots without engaging in reproductive activities; and the trunk-boring stage (January of the following year), during which adults construct galleries and oviposit within tree trunks. To minimize temporal heterogeneity, all specimens from each life stage were collected within a one-week window centered at the midpoint of the respective period.

All infested plant materials were sourced from mature, naturally regenerated *Pinus yunnanensis* (mean DBH of 18–25 cm) within a Yunnan pine-dominated stand. To obtain trunk-boring stage adults, the main stems were cut into 100 cm logs, sealed at both ends with paraffin to prevent desiccation, and placed individually in 80-mesh insect-proof nets (Cui et al., 2023). To obtain shoot-boring stage adults, Yunnan pine shoots with beetle boreholes extending 3–5 cm into the pith were collected and similarly placed in 80-mesh insect-proof nets (Cui et al., 2023). Subsequent identification of adult *T. brevipilosus* relied on several morphological markers, including the presence of granules or punctures on the second interstria along the elytral declivity, as well as the length of interstrial hairs and the hairs originating from punctures (Cui et al., 2023). Sex determination between females and males was based on the shape (semicircular versus rectangular) and size (large versus small) of the last abdominal tergites. Moreover, in living individuals, the male stridulation apparatus produces audible chirps that allow for non-lethal sexing (Cui et al., 2023). Finally, live beetles of each stage and each sex identified in this study were temporarily stored in vials in a refrigerator at 4 °C.

### 2.2 Library construction and high-throughput sequencing of 16S rRNA genes

For each stage, adults were sexed. For each stage-sex combination, three biological replicates were established, each comprising 20 individuals. Replicates for the trunk-boring stage originated from three independent, randomly selected infested trunks, whereas those for the shoot-boring stage were obtained from three independent, randomly selected infested shoots, each from a distinct tree. For each replicate, 20 adults were pooled, and the six gut compartments (foregut, anterior midgut, posterior midgut, ileum, colon, and rectum) and the attached Malpighian tubules (excretory organs)-collectively referred to as the seven intestinal segments hereafter for simplicity-were dissected from the same pooled sample under a stereomicroscope on ice, yielding paired compartment samples within each replicate.

All dissections were performed within 12 h of field collection; when immediate processing was not possible, specimens were stored in sterile vials at 4 °C for no longer than 24 h. To minimize batch-to-batch variation, all dissections were carried out sequentially in a single batch. Prior to dissection, specimens were surface-sterilized by sequential immersion in 75% ethanol for 90 s and 1% sodium hypochlorite for 1 min, followed by five washes with sterile double-distilled H₂O. The intestinal tissue was aseptically excised from the sampled specimens and subsequently divided into three anatomical regions: the foregut, midgut, and hindgut. In parallel, the Malpighian tubules, being the primary excretory organs connected to the intestine, were carefully dissected out. To subdivide the midgut, we observed its autofluorescence under a fluorescence stereomicroscope; the posterior portion consistently exhibited strong blue-green autofluorescence (likely derived from plant-derived ingested materials or digestive residues), whereas the anterior portion showed no detectable fluorescence. This natural autofluorescence, without any exogenous staining, allowed us to reliably separate the midgut into two parts (posterior fluorescent vs. anterior non-fluorescent). Notably, such autofluorescence is commonly observed in the guts of many insect species (Badgaa et al., 2015; Soldano et al., 2026). For the hindgut, we further dissected it into three morphologically distinct regions-ileum, colon, and rectum-based on external constrictions and differences in wall thickness and diameter (Rubio and Acuña, 2006; Candan et al., 2020; Koçakoğlu et al., 2020). To prevent cross-contamination of gut contents between adjacent segments, we adopted a fine-thread ligation method specifically tailored for these small-sized beetles. All dissections were performed under a stereomicroscope in chilled sterile phosphate-buffered saline (PBS) on a cooled glass plate. After carefully removing the surrounding tracheae and fat bodies to expose the intact gut tube, we first isolated each segment by gently passing a pre-sterilized 7–0 nylon suture beneath the gut at the natural constriction between two regions (e.g., midgut-hindgut junction, or ileum-colon junction). Using ultrafine forceps, we tied a single loose overhand knot around the gut, then tightened it just enough to occlude the lumen without crushing or tearing the delicate tissue-the tension was adjusted visually so that the gut wall dimpled slightly but remained intact. A second knot was placed immediately proximal to the first to secure the ligature. After ligating both ends of a given segment (i.e., tying off the connections between adjacent segments), we used a micro-scalpel to cut the gut tube precisely between the two ligatures, leaving both cut ends sealed by the knots. This ensured that no luminal contents could leak out during or after severing. The ligated segment was then transferred intact into a pre-labelled microcentrifuge tube using a sterile pipette tip, without any rinsing or flushing, to preserve the original microbiota. For each new segment, fresh pre-sterilized suture threads and new forceps were used; all instruments (forceps, scalpel, scissors) were flame-sterilized and rinsed in 70% ethanol followed by sterile PBS between every cut and before handling a different specimen. This rigorous protocol effectively eliminated cross-contamination while accommodating the technical challenge of working with minute insects. All dissections were completed within 72 h of collection. The dissected samples were flash-frozen in liquid nitrogen and stored at −80 °C until DNA extraction.

Using E.Z.N.A.® Tissue DNA Kit (Omega), the total microbial DNAs were extracted from the collected samples according to the manufacturer’s instructions. The concentration and purity of the extracted DNA were determined using a NanoDrop 2000 UV–Vis spectrophotometer (Thermo Fisher Scientific), and the qualities of the genomic DNAs were tested by 1% agarose gel electrophoresis. Then, using the extracted DNA as a template, the V3-V4 hypervariable regions of the 16S rRNA gene were amplified by PCR with barcode-containing forward primer 338F (5′-ACTCCTACGGGAGGCAGCAG-3′) and reverse primer 806R (5′-GGACTACHVGGGTWTCTAAT-3′) (Liu et al., 2015). The thermal cycling program was as follows: initial denaturation at 95 °C for 3 min, followed by 28 cycles of 95 °C for 30 s, 55 °C for 30 s, and 72 °C for 30 s, with a final extension at 72 °C for 10 min and holding at 4 °C (PCR instrument: T100 Thermal Cycler, BIO-RAD, USA). The PCR reaction system (20 μL) consisted of 4 μL of 5 × TransStart FastPfu buffer, 2 μL of 2.5 mM dNTPs, 0.8 μL each of forward and reverse primers (5 μM), 0.4 μL of TransStart FastPfu DNA polymerase, and 10 ng of template DNA, made up to 20 μL with nuclease-free water. The PCR products were approximately 700 bp in length and were recovered by 2% agarose gel electrophoresis, purified using a DNA gel recovery purification kit (PCR Clean-Up Kit, Yuhua, China), and quantified with a Qubit 4.0 fluorometer (Thermo Fisher Scientific, USA). To monitor for potential contamination, we prepared a mixture (at equal volume ratios) of all extraction kit reagents (buffers and solutions) and the nuclease-free water used in the bacterial 16S PCR amplification; this mixture served as the negative control (CK) and was subjected to PCR and electrophoresis under identical conditions, and the resulting products showed no visible bands on a 2% agarose gel, confirming the absence of detectable reagent-borne contamination (Supplementary Figure S5). The purified PCR products were used for library construction with the NEXTFLEX Rapid DNA-Seq Kit, which involved (1) adapter ligation, (2) magnetic bead-based selection to remove adapter-dimers, (3) enrichment of library templates by PCR amplification, and (4) magnetic bead recovery of PCR products to obtain the final library. Sequencing was performed on the Illumina NextSeq 2000 platform (2 × 300 bp) by Majorbio Bio-Pharm Technology Co., Ltd., Shanghai, China. A total of 2,825,402 raw paired-end reads were obtained from the trunk-boring stage samples and 3,646,160 from the shoot-boring stage samples (Raw_Reads column in the Supplementary Tables S49, S50). The raw sequence reads were submitted to the NCBI Sequence Read Archive (SRA) database under accession number PRJNA1451412. The dataset can be accessed at: https://www.ncbi.nlm.nih.gov/bioproject/PRJNA1451412/.

The obtained raw reads were processed through the DADA2 pipeline (v1.22.0) (Callahan et al., 2016). Primer and adapter sequences were first removed using cutadapt (v4.0), followed by quality filtering with DADA2’s “filterAndTrim” function using parameters truncQ = 0 and maxEE = 5 (i.e., filtering based on maximum expected errors rather than fixed-length truncation), error correction, and amplicon sequence variant (ASV) inference. Downstream analyses and data handling were performed using the Phyloseq package (v1.38.0) (McMurdie and Holmes, 2013) within the RStudio environment (v4.1.2). Following quality filtering and primer/adapter removal, retained read counts ranged from 41,764 to 73,910 for trunk-boring and from 49,881 to 281,151 for shoot-boring samples (Filtered_Reads column in the Supplementary Tables S49, S50). Following DADA2 denoising, paired-end merging, and stringent chimera removal, effective read counts ranged from 37,918 to 70,007 (mean: ~55,000) for trunk-boring samples and from 40,638 to 245,722 (mean: ~65,000) for shoot-boring samples (NonChimeric_Reads in Supplementary Tables S49, S50). As an additional *in silico* quality-control measure, we applied abundance-based filtering to exclude ASVs with a total read count of fewer than 10 across the entire dataset prior to downstream analyses. After this filtering, the number of ASVs per sample ranged from 26 to 237 (mean: ~71) for the trunk-boring stage and from 19 to 371 (mean: ~101) for the shoot-boring stage (ASVs_num in the Supplementary Tables S49, S50) in the updated dataset.

To ensure data fidelity, we rigorously filtered the ASV table to remove any ASVs annotated to Eukaryota (host origin), Chloroplast, or Mitochondria, retaining only those confidently classified as Bacteria (at the phylum level and below) for all subsequent community analyses. Taxonomic classification of ASVs was achieved via a naive Bayesian classifier referencing the SILVA nr99 v.138 database (Wickham, 2016). To illustrate the distribution of bacterial taxa across different hierarchical levels (from phylum to genus), a heatmap was generated utilizing the ggplot2 package (v3.3.5) (Quast et al., 2012). Bioinformatic characterization of the gut bacteria was performed on the Majorbio Cloud platform^1^, leveraging representative sequences derived from ASVs.

### 2.3 Functional predictions of the bacterial community

To infer the functional potential of the gut bacterial communities in *T. brevipilosus*, we used PICRUSt2 (v2.2.0) on the amplicon sequence variant (ASV) table derived from each intestinal section (Douglas et al., 2020). The workflow first normalized ASV abundances by predicted 16S rRNA gene copy numbers, then predicted metagenomic functions against the KEGG Orthology database under default parameters. To assess prediction reliability, we calculated the weighted Nearest Sequenced Taxon Index (NSTI) for every ASV. Functional profiles were subsequently summarized at KEGG pathway Level 2, and the resulting relative abundances, together with their pairwise Spearman correlations, were visualized as heatmaps generated with the heatmap and corrplot packages in R.

### 2.4 Statistical analysis

Statistical analyses were performed using R software (version 4.2.1) and SPSS for Windows (version 26.0). To account for the paired experimental design-wherein the six gut compartments (foregut, anterior midgut M1, posterior midgut M2, and hindgut subsections ileum H1, colon H2, and rectum H3) and the attached Malpighian tubules were dissected from the same pooled sample of 20 adults per replicate, and replicates originated from independent source trees (trunks for the trunk-boring stage and shoots for the shoot-boring stage).

#### 2.4.1 Alpha diversity

Alpha-diversity indices, including Sobs, Ace, Chao, Shannon, Simpson and Pielou’s evenness, were calculated from rarefied ASV tables using QIIME (v1.9.1). To formally evaluate the effects of gut compartment and its interactions with sex and feeding stage on alpha-diversity metrics, we fitted linear mixed models (LMMs) using SPSS (version 26.0). Specifically, to examine sex-specific compartmentalization patterns, we performed separate LMMs for the trunk-boring and shoot-boring stages, with “compartment,” “sex” and their interaction as fixed factors and “replicate” as a random intercept. To examine stage-dependent shifts in compartmentalization, we performed separate LMMs for males and females, with “compartment,” “stage” and their interaction as fixed factors and “replicate” as a random intercept. In all models, replicate was included as a random intercept term to account for the repeated-measures structure (i.e., multiple compartments from the same pooled sample). When the omnibus *F*-test for a given interaction term was globally significant (*p* < 0.05), we performed post-hoc pairwise comparisons using estimated marginal means in SPSS, with *p*-values adjusted via the Bonferroni correction to control the family-wise error rate.

#### 2.4.2 Beta diversity

For beta-diversity analysis, we computed Bray-Curtis dissimilarities from the same rarefied ASV data and visualized community structure using principal coordinate analysis (PCoA). Overall microbial community differences among compartments were evaluated using permutational multivariate analysis of variance (PERMANOVA, 999 permutations) as implemented in the adonis2 function of the R package vegan (v 3.3.1). Importantly, permutation tests were explicitly stratified by replicate (strata = replicate) to restrict random permutations within each biological replicate, thereby preserving the paired structure of the compartment samples.

#### 2.4.3 Differential abundance analysis of microbial taxa

Differential abundance of microbial taxa at phylum to genus levels was assessed using a combination of primary and sensitivity analyses. As the primary approach, we employed MaAsLin2 (Microbiome Multivariate Association with Linear Models 2), a multivariate linear modelling framework specifically designed for microbiome association studies that accounts for compositional data effects through total-sum scaling (TSS) normalization followed by log transformation (Mallick et al., 2021). The model was fitted with Group (seven intestinal compartments: F, M1, M2, Mt., H1, H2, and H3) as the fixed effect. To perform exhaustive pairwise comparisons across all segments without assigning a reference category, we specified pairwise contrasts for the Group variable; accordingly, a positive coefficient indicates that the former group in the comparison has a higher abundance, while a negative coefficient indicates the opposite. Features were standardized (standardize = TRUE) to allow comparability of coefficients. A prevalence filter was applied, retaining only taxa detected in at least 10% of all samples (min_prevalence = 0.1). The analysis was run using the linear model (method = “LM”) with log transformation (transform = “LOG”) and default minimum abundance (min_abundance = 0.0). The maximum significance threshold for output was set to max_significance = 0.25. To control the false discovery rate (FDR), we applied the Benjamini-Hochberg (BH) procedure to all raw *p*-values, and all reported *p*-values in the main text are adjusted *q*-values. Statistical significance was defined at *q* < 0.25, as recommended for exploratory microbiome studies by the MaAsLin2 developers. Tables 1–4 summarize all significant associations with *q*-values below this cutoff.

**Table 1:** MaAsLin2 analysis of differentially abundant bacterial taxa (phylum to genus) among seven gut segments (F, M1, M2, Mt., H1, H2, H3) of female *Tomicus brevipilosus* during the trunk-boring stage.

Level	Taxon	Comparison	coef	qval
Phylum	Actinomycetota	BF_H3_vs_BF_M1	1.8383	0.1704
		BF_F_vs_BF_Mt	−2.4617	0.1726
		BF_F_vs_BF_H3	−1.6163	0.1993
		BF_M1_vs_BF_Mt	−2.6837	0.237
	Bacillota	BF_F_vs_BF_Mt	−5.0458	0.1726
		BF_F_vs_BF_H3	−5.5878	0.1993
	Pseudomonadota	BF_F_vs_BF_H3	0.9305	0.1993
		BF_F_vs_BF_Mt	0.4829	0.2028
Class	Actinobacteria	BF_F_vs_BF_Mt	−2.4614	0.1728
		BF_F_vs_BF_H3	−1.6143	0.2434
	Acidimicrobiia	BF_F_vs_BF_H2	−2.5554	0.2195
	Bacilli	BF_F_vs_BF_Mt	−5.1632	0.1728
	Clostridia	BF_F_vs_BF_Mt	−2.3518	0.1257
		BF_F_vs_BF_H2	−2.606	0.2195
		BF_F_vs_BF_H3	−1.9358	0.2434
	Gammaproteobacteria	BF_F_vs_BF_H3	1.5065	0.2434
Order	Bacillales	BF_F_vs_BF_H3	−2.2172	0.1562
		BF_H3_vs_BF_M1	2.1771	0.2428
	Lachnospirales	BF_F_vs_BF_H3	−1.7525	0.1562
	Mycobacteriales	BF_H3_vs_BF_M1	1.8525	0.2428
	Nitriliruptorales	BF_H3_vs_BF_M1	1.8793	0.2428
	Pseudomonadales	BF_H3_vs_BF_M1	2.2897	0.2428
Family	Mycobacteriaceae	BF_H3_vs_BF_M1	1.8835	0.0954
		BF_M1_vs_BF_Mt	−2.9904	0.2444
	Halomonadaceae	BF_H3_vs_BF_M1	2.3748	0.2227
		BF_M1_vs_BF_Mt	−2.92	0.2444
	Nitriliruptoraceae	BF_H3_vs_BF_M1	1.8793	0.2227
		BF_M1_vs_BF_Mt	−2.5298	0.2444
	Alcaligenaceae	BF_M1_vs_BF_Mt	−2.7229	0.2444
	Comamonadaceae	BF_M1_vs_BF_Mt	−3.1991	0.2444
	Dietziaceae	BF_M1_vs_BF_Mt	−2.662	0.2444
	Micrococcaceae	BF_M1_vs_BF_Mt	−2.7494	0.2444
	Moraxellaceae	BF_M1_vs_BF_Mt	−4.0462	0.2444
	Salisediminibacteriaceae	BF_M1_vs_BF_Mt	−3.5187	0.2444
Genus	*Delftia*	BF_H3_vs_BF_Mt	3.3063	0.0846
		BF_H3_vs_BF_M1	1.1802	0.1278
		BF_F_vs_BF_H3	−1.1802	0.2079
	*Halomonas*	BF_H3_vs_BF_M1	2.3748	0.2273
	*Alteribacter*	BF_F_vs_BF_H3	−1.9305	0.2383

A positive coefficient (coef) indicates that the former group in the comparison has a higher abundance, while a negative coefficient (coef) indicates the opposite. BF = trunk-boring female, F = foregut, M1 = anterior midgut, M2 = posterior midgut, Mt = Malpighian tubules, H1 = ileum, H2 = colon, H3 = rectum.

**Table 2:** MaAsLin2 analysis of differentially abundant bacterial taxa (phylum to genus) among seven gut segments (F, M1, M2, Mt., H1, H2, H3) of male *Tomicus brevipilosus* during the trunk-boring stage.

Level	Taxon	Comparison	coef	qval
Phylum	Bacillota	BM_M1_vs_BM_Mt	−3.7917	0.0011
		BM_H3_vs_BM_M1	8.128	0.0065
		BM_F_vs_BM_H3	−8.6554	0.016
		BM_F_vs_BM_Mt	−4.3191	0.0171
		BM_F_vs_BM_H1	−5.5013	0.0344
		BM_H1_vs_BM_M1	4.9739	0.0367
		BM_H2_vs_BM_M1	4.8127	0.0692
		BM_F_vs_BM_H2	−5.3401	0.0746
		BM_H3_vs_BM_Mt	4.3363	0.0795
	Actinomycetota	BM_M1_vs_BM_Mt	−4.1455	0.0029
		BM_F_vs_BM_Mt	−4.9164	0.0114
		BM_H3_vs_BM_M1	3.8538	0.0142
		BM_F_vs_BM_H3	−4.6247	0.0205
		BM_F_vs_BM_H1	−4.6938	0.0344
		BM_H1_vs_BM_M1	3.9229	0.0367
		BM_H2_vs_BM_M1	4.1617	0.0692
		BM_F_vs_BM_H2	−4.9326	0.0746
	Pseudomonadota	BM_F_vs_BM_Mt	0.8418	0.087
		BM_M1_vs_BM_Mt	0.8279	0.1119
		BM_F_vs_BM_H2	1.7999	0.2341
		BM_F_vs_BM_H3	2.1489	0.2376
		BM_H3_vs_BM_M1	−2.135	0.2421
	Bacteroidota	BM_F_vs_BM_Mt	−2.4783	0.2015
		BM_F_vs_BM_H2	−5.966	0.2341
Class	Actinobacteria	BM_M1_vs_BM_Mt	−4.1448	0.0039
		BM_F_vs_BM_Mt	−4.9146	0.0165
		BM_H3_vs_BM_M1	3.8411	0.0179
		BM_F_vs_BM_H3	−4.6108	0.0381
		BM_F_vs_BM_H1	−4.6877	0.0462
		BM_H1_vs_BM_M1	3.9179	0.0528
		BM_H2_vs_BM_M1	4.1597	0.0979
		BM_F_vs_BM_H2	−4.9295	0.1117
	Bacilli	BM_M1_vs_BM_Mt	−3.9999	0.0039
		BM_H3_vs_BM_M1	5.8423	0.004
		BM_F_vs_BM_Mt	−4.2466	0.0264
		BM_F_vs_BM_H3	−6.089	0.0374
		BM_H1_vs_BM_M1	5.0248	0.0452
		BM_F_vs_BM_H1	−5.2715	0.0462
		BM_H3_vs_BM_Mt	1.8424	0.0978
		BM_H2_vs_BM_M1	4.8702	0.0979
		BM_F_vs_BM_H2	−5.1169	0.1117
	Clostridia	BM_H3_vs_BM_M1	10.408	0.0079
		BM_F_vs_BM_M1	−1.585	0.0842
		BM_H3_vs_BM_Mt	8.8764	0.0885
		BM_F_vs_BM_Mt	−2.5316	0.1218
		BM_H3_vs_BM_M2	8.7515	0.1769
		BM_F_vs_BM_H2	−5.137	0.2053
		BM_F_vs_BM_H1	−4.1631	0.2139
		BM_F_vs_BM_H3	−2.7878	0.236
	Acidimicrobiia	BM_M1_vs_BM_Mt	−3.7542	0.0141
		BM_F_vs_BM_Mt	−1.9176	0.0481
		BM_H2_vs_BM_M1	4.4171	0.1534
		BM_F_vs_BM_H1	−3.0676	0.193
		BM_F_vs_BM_H2	−3.4285	0.197
		BM_H1_vs_BM_M1	3.3193	0.2325
		BM_F_vs_BM_H3	−3.537	0.236
	Alphaproteobacteria	BM_M1_vs_BM_Mt	−3.1477	0.0185
		BM_F_vs_BM_Mt	−4.6545	0.0363
		BM_F_vs_BM_H1	−3.591	0.193
		BM_F_vs_BM_H2	−3.6701	0.197
		BM_F_vs_BM_H3	−3.5415	0.236
	Gammaproteobacteria	BM_F_vs_BM_Mt	0.8542	0.0767
		BM_M1_vs_BM_Mt	0.84	0.0932
		BM_F_vs_BM_H1	1.0898	0.2139
		BM_F_vs_BM_H2	1.8242	0.2144
		BM_F_vs_BM_H3	2.1692	0.236
	Bacteroidia	BM_F_vs_BM_Mt	−2.4783	0.1663
		BM_F_vs_BM_H2	−5.966	0.2053
		BM_F_vs_BM_H1	−4.0384	0.2335
		BM_F_vs_BM_H3	−2.8621	0.2464
	Coriobacteriia	BM_F_vs_BM_H3	−3.0428	0.236
		BM_F_vs_BM_H2	−0.7826	0.2496
Order	Mycobacteriales	BM_M1_vs_BM_Mt	−4.5937	0.0036
		BM_F_vs_BM_Mt	−5.182	0.0211
		BM_H3_vs_BM_M1	4.4919	0.0563
		BM_H1_vs_BM_M1	4.8572	0.0565
		BM_F_vs_BM_H1	−5.4455	0.089
		BM_F_vs_BM_H2	−5.7677	0.1326
		BM_H2_vs_BM_M1	5.1794	0.1919
		BM_F_vs_BM_H3	−5.0803	0.2101
	Sphingomonadales	BM_M1_vs_BM_Mt	−4.607	0.0036
		BM_F_vs_BM_Mt	−1.3158	0.0211
		BM_F_vs_BM_H1	−3.0217	0.1644
		BM_H1_vs_BM_M1	3.4254	0.1704
	Streptosporangiales	BM_M1_vs_BM_Mt	−3.696	0.0036
		BM_F_vs_BM_Mt	−1.3611	0.0211
		BM_H1_vs_BM_M1	4.0149	0.0565
		BM_F_vs_BM_M1	−1.384	0.064
		BM_F_vs_BM_H1	−2.1765	0.089
		BM_H3_vs_BM_M1	3.5466	0.0918
		BM_F_vs_BM_H2	−2.9849	0.1969
		BM_H2_vs_BM_M1	3.6597	0.2139
		BM_F_vs_BM_H3	−1.6329	0.2392
	Bacillales	BM_M1_vs_BM_Mt	−4.1492	0.0174
		BM_F_vs_BM_Mt	−4.2019	0.0282
		BM_H1_vs_BM_M1	5.2053	0.0565
		BM_F_vs_BM_H1	−5.258	0.089
		BM_H3_vs_BM_M1	4.7512	0.0918
		BM_F_vs_BM_H2	−5.0634	0.1646
		BM_H2_vs_BM_M1	5.0107	0.2139
		BM_F_vs_BM_H3	−4.8038	0.222
	Micrococcales	BM_M1_vs_BM_Mt	−3.8031	0.0174
		BM_F_vs_BM_Mt	−4.6435	0.0211
		BM_F_vs_BM_H1	−4.051	0.089
		BM_H3_vs_BM_M1	2.7824	0.1001
		BM_H1_vs_BM_M1	3.2105	0.106
		BM_F_vs_BM_H2	−4.2639	0.1646
		BM_H2_vs_BM_M1	3.4235	0.2139
		BM_F_vs_BM_H3	−3.6229	0.222
	Microtrichales	BM_M1_vs_BM_Mt	−3.7542	0.0174
		BM_F_vs_BM_Mt	−1.9176	0.0576
		BM_F_vs_BM_H1	−3.0676	0.1644
		BM_H1_vs_BM_M1	3.3193	0.1704
		BM_F_vs_BM_H2	−3.2146	0.1969
		BM_H2_vs_BM_M1	4.2032	0.2139
		BM_F_vs_BM_H3	−3.537	0.2338
	Nitriliruptorales	BM_M1_vs_BM_Mt	−3.8806	0.0205
		BM_F_vs_BM_Mt	−4.6573	0.0264
		BM_F_vs_BM_H1	−3.7536	0.089
		BM_H1_vs_BM_M1	2.9769	0.1274
		BM_F_vs_BM_H2	−3.571	0.1969
		BM_H3_vs_BM_M1	2.7632	0.2191
		BM_F_vs_BM_H3	−3.5399	0.222
	Burkholderiales	BM_F_vs_BM_Mt	−4.823	0.0211
		BM_M1_vs_BM_Mt	−2.5431	0.0291
		BM_F_vs_BM_H1	−4.2867	0.089
		BM_F_vs_BM_H2	−3.947	0.1969
		BM_F_vs_BM_H3	−3.6879	0.222
	Pseudomonadales	BM_F_vs_BM_Mt	−4.3854	0.0478
		BM_M1_vs_BM_Mt	−3.6119	0.0713
		BM_F_vs_BM_H1	−3.2316	0.089
		BM_H1_vs_BM_M1	2.458	0.1704
		BM_F_vs_BM_H2	−3.4299	0.1969
		BM_H3_vs_BM_M1	2.1159	0.2191
		BM_F_vs_BM_H3	−2.8895	0.222
	Lachnospirales	BM_H3_vs_BM_M1	10.3128	0.0378
		BM_H3_vs_BM_Mt	10.6461	0.0503
		BM_H3_vs_BM_M2	6.7233	0.1054
		BM_F_vs_BM_H1	−4.3292	0.2157
		BM_F_vs_BM_H3	−2.971	0.222
	Oscillospirales	BM_H3_vs_BM_M1	8.8396	0.0378
		BM_H3_vs_BM_M2	7.8396	0.1054
		BM_H3_vs_BM_Mt	6.5508	0.199
		BM_F_vs_BM_H3	−2.6711	0.222
	Peptostreptococcales. Tissierellales	BM_H3_vs_BM_M2	7.9641	0.0237
		BM_H1_vs_BM_H3	−6.3345	0.0419
		BM_H3_vs_BM_Mt	5.5491	0.0503
		BM_F_vs_BM_H1	−1.548	0.089
		BM_H1_vs_BM_M1	1.548	0.1188
		BM_H3_vs_BM_M1	1.9341	0.2191
		BM_F_vs_BM_H3	−1.9341	0.222
		BM_H2_vs_BM_H3	−3.9912	0.2406
	Clostridiales	BM_H1_vs_BM_H3	−5.4084	0.0419
		BM_H2_vs_BM_H3	−4.6308	0.1495
		BM_H3_vs_BM_M1	1.7036	0.2191
		BM_F_vs_BM_H3	−1.7036	0.222
	Lactobacillales	BM_H3_vs_BM_M1	5.8988	0.0918
		BM_F_vs_BM_H1	−3.2701	0.1658
		BM_F_vs_BM_H3	−3.0097	0.2324
	Hyphomicrobiales	BM_F_vs_BM_Mt	−3.8524	0.198
	Enterobacterales	BM_H3_vs_BM_M1	−2.5484	0.2191
		BM_F_vs_BM_H3	2.5718	0.222
		BM_F_vs_BM_H1	1.3586	0.2433
	Lysobacterales	BM_M1_vs_BM_Mt	2.5752	0.1984
		BM_H3_vs_BM_M1	−3.056	0.2191
		BM_F_vs_BM_H3	−2	0.222
	Bacteroidales	BM_F_vs_BM_H1	−2.0738	0.089
		BM_H1_vs_BM_M1	2.0738	0.1188
	Propionibacteriales	BM_F_vs_BM_H2	−4.7861	0.1326
		BM_H3_vs_BM_M1	2.7543	0.2191
		BM_F_vs_BM_H3	−4.158	0.222
	Euzebyales	BM_F_vs_BM_H1	−2.1555	0.089
		BM_H1_vs_BM_M1	2.9742	0.106
		BM_F_vs_BM_H2	−2.4002	0.1969
	Erysipelotrichales	BM_F_vs_BM_H3	−4.6051	0.222
		BM_H3_vs_BM_M1	3.732	0.2443
	Coriobacteriales	BM_H3_vs_BM_M1	3.1265	0.2191
		BM_F_vs_BM_H3	−3.1265	0.222
	Bifidobacteriales	BM_H3_vs_BM_M1	2.6604	0.2191
		BM_F_vs_BM_H3	−2.6604	0.222
	Sphingobacteriales	BM_H3_vs_BM_M1	1.4951	0.2191
		BM_F_vs_BM_H3	−1.4951	0.222
Family	Dietziaceae	BM_M1_vs_BM_Mt	−4.587	0.006
		BM_F_vs_BM_Mt	−5.1843	0.0182
		BM_H1_vs_BM_M1	4.8584	0.0577
		BM_H3_vs_BM_M1	4.4913	0.0703
		BM_F_vs_BM_H1	−5.4557	0.08
		BM_F_vs_BM_H2	−5.6716	0.1476
		BM_F_vs_BM_H3	−5.0887	0.1682
		BM_H2_vs_BM_M1	5.0742	0.2245
	Moraxellaceae	BM_M1_vs_BM_Mt	−4.7689	0.0088
		BM_F_vs_BM_Mt	−4.5308	0.0182
		BM_H3_vs_BM_M1	4.2818	0.0565
		BM_H1_vs_BM_M1	4.9717	0.0577
		BM_F_vs_BM_H1	−4.7336	0.08
		BM_F_vs_BM_H3	−4.0436	0.1467
		BM_F_vs_BM_H2	−6.0719	0.2034
		BM_H2_vs_BM_M1	6.31	0.2245
	Mycobacteriaceae	BM_M1_vs_BM_Mt	−4.737	0.014
		BM_F_vs_BM_Mt	−5.347	0.0553
		BM_H2_vs_BM_M1	5.8735	0.0745
		BM_H1_vs_BM_M1	4.8242	0.0943
		BM_H3_vs_BM_M1	4.5613	0.1003
		BM_F_vs_BM_H1	−5.4342	0.1464
		BM_F_vs_BM_H2	−6.4835	0.1476
		BM_F_vs_BM_H3	−5.1713	0.2052
	Comamonadaceae	BM_M1_vs_BM_Mt	−4.0397	0.0146
		BM_F_vs_BM_Mt	−3.6247	0.0258
		BM_H1_vs_BM_M1	4.716	0.0577
		BM_F_vs_BM_H1	−4.301	0.08
		BM_H3_vs_BM_M1	4.5362	0.1091
		BM_F_vs_BM_H3	−4.1212	0.1963
		BM_F_vs_BM_H2	−4.2534	0.2466
	Salisediminibacteriaceae	BM_M1_vs_BM_Mt	−3.94	0.0191
		BM_F_vs_BM_Mt	−6.1233	0.0353
		BM_H1_vs_BM_M1	5.236	0.0577
		BM_F_vs_BM_H1	−7.4193	0.08
		BM_H3_vs_BM_M1	4.7652	0.1091
		BM_F_vs_BM_H2	−7.2409	0.1476
		BM_F_vs_BM_H3	−6.9484	0.1963
		BM_H2_vs_BM_M1	5.0577	0.2245
	Micrococcaceae	BM_M1_vs_BM_Mt	−3.8233	0.0191
		BM_F_vs_BM_Mt	−4.6376	0.0258
		BM_H1_vs_BM_M1	3.2244	0.0943
		BM_H3_vs_BM_M1	2.7837	0.1116
		BM_F_vs_BM_H1	−4.0387	0.1375
		BM_F_vs_BM_H3	−3.598	0.1963
		BM_F_vs_BM_H2	−4.2461	0.2219
	Nitriliruptoraceae	BM_M1_vs_BM_Mt	−3.8806	0.0221
		BM_F_vs_BM_Mt	−4.6573	0.0339
		BM_F_vs_BM_H1	−3.7536	0.1464
		BM_H1_vs_BM_M1	2.9769	0.1702
		BM_F_vs_BM_H3	−3.5399	0.2497
	Nocardiopsidaceae	BM_M1_vs_BM_Mt	−3.696	0.006
		BM_F_vs_BM_Mt	−1.3611	0.0249
		BM_H1_vs_BM_M1	4.0149	0.0577
		BM_H3_vs_BM_M1	3.5466	0.1091
		BM_F_vs_BM_M1	−1.384	0.1135
		BM_F_vs_BM_H1	−2.1765	0.1464
	Oxalobacteraceae	BM_M1_vs_BM_Mt	−4.332	0.0085
		BM_F_vs_BM_Mt	−5.9169	0.0099
		BM_F_vs_BM_H1	−4.2729	0.1464
		BM_F_vs_BM_H2	−3.6882	0.1476
		BM_F_vs_BM_H3	−2.8873	0.2497
	Sphingomonadaceae	BM_M1_vs_BM_Mt	−4.607	0.006
		BM_F_vs_BM_Mt	−1.3158	0.0258
		BM_F_vs_BM_H1	−3.0217	0.218
		BM_H1_vs_BM_M1	3.4254	0.2258
	Halomonadaceae	BM_F_vs_BM_Mt	−4.4353	0.0649
		BM_M1_vs_BM_Mt	−3.6015	0.0975
		BM_F_vs_BM_H1	−3.0805	0.1464
		BM_H1_vs_BM_M1	2.2467	0.2315
		BM_F_vs_BM_H3	−2.7476	0.2497
	Pseudomonadaceae	BM_F_vs_BM_Mt	−4.1749	0.0684
		BM_M1_vs_BM_Mt	−3.346	0.0975
		BM_F_vs_BM_H1	−3.1648	0.2083
		BM_F_vs_BM_H3	−2.9426	0.2497
	Iamiaceae	BM_M1_vs_BM_Mt	−3.7542	0.0191
		BM_F_vs_BM_Mt	−1.9176	0.0684
		BM_F_vs_BM_H1	−3.0676	0.218
		BM_H1_vs_BM_M1	3.3193	0.2258
		BM_F_vs_BM_H3	−3.537	0.2497
	Lactobacillaceae	BM_F_vs_BM_H3	−1.7037	0.1963
		BM_H3_vs_BM_Mt	1.7037	0.2319
	Xanthobacteraceae	BM_F_vs_BM_H1	−2.6025	0.2083
		BM_H1_vs_BM_M1	2.6025	0.2258
	Bacteroidaceae	BM_H1_vs_BM_M1	1.4688	0.0943
		BM_F_vs_BM_H1	−1.4688	0.1451
		BM_F_vs_BM_H3	−2.3697	0.2497
	Bogoriellaceae	BM_F_vs_BM_H1	−2.7052	0.2001
		BM_H1_vs_BM_M1	2.7052	0.2224
		BM_F_vs_BM_H3	−2.0148	0.2497
	Coriobacteriaceae	BM_H3_vs_BM_M1	1.5087	0.1881
		BM_F_vs_BM_H3	−1.5087	0.2052
	Euzebyaceae	BM_H1_vs_BM_M1	2.9742	0.0943
		BM_F_vs_BM_H1	−2.1555	0.1464
	X. Eubacterium._coprostanoligenes_group	BM_H3_vs_BM_M1	1.4898	0.1091
		BM_F_vs_BM_H3	−1.4898	0.1963
		BM_H3_vs_BM_Mt	1.4898	0.2298
	Ruminococcaceae	BM_H3_vs_BM_M1	8.5561	0.0703
		BM_H3_vs_BM_Mt	5.2205	0.2319
		BM_F_vs_BM_H3	−2.9688	0.2497
	Lachnospiraceae	BM_H3_vs_BM_M1	10.3128	0.0565
		BM_H3_vs_BM_Mt	10.6461	0.0902
		BM_F_vs_BM_H3	−2.971	0.2497
	Oscillospiraceae	BM_H3_vs_BM_Mt	3.7401	0.1259
		BM_H3_vs_BM_M1	1.8407	0.1881
		BM_H1_vs_BM_H3	−3.2546	0.2207
		BM_F_vs_BM_H3	−1.8407	0.2052
Genus	*Acinetobacter*	BM_M1_vs_BM_Mt	−4.6781	0.0129
		BM_F_vs_BM_Mt	−4.44	0.0226
		BM_H1_vs_BM_M1	4.9717	0.0733
		BM_F_vs_BM_H1	−4.7336	0.0773
		BM_H3_vs_BM_M1	4.2818	0.1949
	*Alteribacter*	BM_M1_vs_BM_Mt	−3.768	0.0418
		BM_F_vs_BM_Mt	−5.8866	0.063
		BM_H1_vs_BM_M1	5.348	0.0733
		BM_F_vs_BM_H1	−7.4666	0.0773
	*Aquihabitans*	BM_M1_vs_BM_Mt	−3.7542	0.0318
		BM_F_vs_BM_Mt	−1.9176	0.0983
	*Bacteroides*	BM_H1_vs_BM_M1	1.4688	0.1128
		BM_F_vs_BM_H1	−1.4688	0.1809
	*Brucella*	BM_H1_vs_BM_M1	−2.5499	0.1128
	*Dietzia*	BM_M1_vs_BM_Mt	−4.587	0.0108
		BM_F_vs_BM_Mt	−5.1843	0.0226
		BM_H1_vs_BM_M1	4.8584	0.0733
		BM_F_vs_BM_H1	−5.4557	0.0773
		BM_H3_vs_BM_M1	4.4913	0.2426
	*Egicoccus*	BM_H1_vs_BM_M1	4.5774	0.1128
	*Halomonas*	BM_F_vs_BM_Mt	−4.4353	0.0983
		BM_M1_vs_BM_Mt	−3.6015	0.1439
		BM_F_vs_BM_H1	−3.0805	0.1966
	*Herbaspirillum*	BM_M1_vs_BM_Mt	−4.332	0.0129
		BM_F_vs_BM_Mt	−5.9169	0.0141
		BM_F_vs_BM_H1	−4.2729	0.1966
	*Lactobacillus*	BM_H1_vs_BM_M1	1.3333	0.0733
		BM_F_vs_BM_H1	−1.3333	0.0773
		BM_H1_vs_BM_M2	1.3333	0.1984
		BM_H1_vs_BM_Mt	1.3333	0.2185
	*Nesterenkonia*	BM_M1_vs_BM_Mt	−3.8209	0.0318
		BM_F_vs_BM_Mt	−4.6352	0.0478
		BM_H1_vs_BM_M1	3.2244	0.1128
		BM_F_vs_BM_H1	−4.0387	0.1667
	*Nitriliruptor*	BM_F_vs_BM_Mt	−1.9415	0.0983
		BM_M1_vs_BM_Mt	−1.9415	0.1216
	*Nocardiopsis*	BM_M1_vs_BM_Mt	−3.696	0.0108
		BM_F_vs_BM_Mt	−1.3611	0.0357
		BM_H1_vs_BM_M1	4.0149	0.0733
		BM_F_vs_BM_M1	−1.384	0.1426
		BM_F_vs_BM_H1	−2.1765	0.1966
	norank_f__Euzebyaceae	BM_H1_vs_BM_M1	2.9742	0.1128
		BM_F_vs_BM_H1	−2.1555	0.1966
	*Pseudomonas*	BM_F_vs_BM_Mt	−4.1749	0.0983
		BM_M1_vs_BM_Mt	−3.346	0.1439
	*Sphingomonas*	BM_M1_vs_BM_Mt	−3.8491	0.0129
		BM_F_vs_BM_Mt	−1.3751	0.055
	unclassified_f__Mycobacteriaceae	BM_M1_vs_BM_Mt	−4.737	0.0206
		BM_F_vs_BM_Mt	−5.347	0.0881
		BM_H1_vs_BM_M1	4.7645	0.1128
		BM_F_vs_BM_H1	−5.3745	0.1966
	unclassified_f__Nitriliruptoraceae	BM_M1_vs_BM_Mt	−4.036	0.0408
		BM_F_vs_BM_Mt	−4.6998	0.0478
		BM_F_vs_BM_H1	−3.7524	0.1966
		BM_H1_vs_BM_M1	3.0887	0.2085
	*Variovorax*	BM_F_vs_BM_H1	−4.0158	0.0773
		BM_H1_vs_BM_M1	1.7053	0.1128

A positive coefficient (coef) indicates that the former group in the comparison has a higher abundance, while a negative coefficient (coef) indicates the opposite. BM = trunk-boring male, F = foregut, M1 = anterior midgut, M2 = posterior midgut, Mt = Malpighian tubules, H1 = ileum, H2 = colon, H3 = rectum.

**Table 3:** MaAsLin2 analysis of differentially abundant bacterial taxa (phylum to genus) among seven gut segments (F, M1, M2, Mt., H1, H2, H3) of female *Tomicus brevipilosus* during the shoot-boring stage.

Level	Taxon	Comparison	coef	qval
Phylum	Actinomycetota	SBF_H1_vs_SBF_H2	4.0556	0.0087
		SBF_H1_vs_SBF_M2	3.7223	0.0463
		SBF_H1_vs_SBF_H3	2.3889	0.0588
		SBF_F_vs_SBF_H1	−1.2628	0.0887
		SBF_H1_vs_SBF_Mt	1.219	0.2497
	Patescibacteria	SBF_F_vs_SBF_H1	−1.3333	0.0887
		SBF_H1_vs_SBF_M2	1.3333	0.0887
		SBF_H1_vs_SBF_H3	1.3333	0.0968
		SBF_H1_vs_SBF_H2	1.3333	0.1048
		SBF_H1_vs_SBF_Mt	1.3333	0.1774
		SBF_H1_vs_SBF_M1	1.3333	0.1936
Class	Actinobacteria	SBF_H1_vs_SBF_H2	4.0974	0.0458
		SBF_H1_vs_SBF_M2	4.0974	0.0458
		SBF_F_vs_SBF_H1	−1.8458	0.1166
		SBF_H1_vs_SBF_H3	2.0974	0.1534
	Clostridia	SBF_F_vs_SBF_H1	−1.195	0.0682
		SBF_H1_vs_SBF_Mt	1.195	0.0682
		SBF_H1_vs_SBF_H3	1.195	0.0718
		SBF_H1_vs_SBF_M1	1.195	0.0718
		SBF_H1_vs_SBF_H2	1.8617	0.0894
		SBF_H1_vs_SBF_M2	1.8617	0.0894
	Parcubacteria	SBF_H1_vs_SBF_H2	1.3333	0.1129
		SBF_H1_vs_SBF_M2	1.3333	0.1129
		SBF_F_vs_SBF_H1	−1.3333	0.1166
		SBF_H1_vs_SBF_Mt	1.3333	0.1532
		SBF_H1_vs_SBF_H3	1.3333	0.1534
		SBF_H1_vs_SBF_M1	1.3333	0.1613
Order	Staphylococcales	SBF_H1_vs_SBF_H2	3.6591	0.07
		SBF_F_vs_SBF_H1	−1.4075	0.2137
		SBF_H1_vs_SBF_M1	1.4075	0.2218
		SBF_H1_vs_SBF_M2	2.6591	0.2258
	Candidatus_Kaiserbacteria	SBF_F_vs_SBF_H1	−1.3333	0.2137
		SBF_H1_vs_SBF_M1	1.3333	0.2218
		SBF_H1_vs_SBF_H2	1.3333	0.2258
		SBF_H1_vs_SBF_M2	1.3333	0.2258
	Frankiales	SBF_F_vs_SBF_H1	−1.8617	0.2137
		SBF_H1_vs_SBF_M1	1.8617	0.2218
		SBF_H1_vs_SBF_H2	1.8617	0.2258
		SBF_H1_vs_SBF_M2	1.8617	0.2258
	Lachnospirales	SBF_F_vs_SBF_H1	−1.3333	0.2137
		SBF_H1_vs_SBF_M1	1.3333	0.2218
		SBF_H1_vs_SBF_H2	1.3333	0.2258
		SBF_H1_vs_SBF_M2	1.3333	0.2258
Family	Staphylococcaceae	SBF_H1_vs_SBF_H2	3.6591	0.1113
Genus	*Staphylococcus*	SBF_H1_vs_SBF_H2	3.6591	0.15

A positive coefficient (coef) indicates that the former group in the comparison has a higher abundance, while a negative coefficient (coef) indicates the opposite. SBF = shoot-boring female, F = foregut, M1 = anterior midgut, M2 = posterior midgut, Mt = Malpighian tubules, H1 = ileum, H2 = colon, H3 = rectum.

**Table 4:** MaAsLin2 analysis of differentially abundant bacterial taxa (phylum to genus) among seven gut segments (F, M1, M2, Mt., H1, H2, H3) of male *Tomicus brevipilosus* during the shoot-boring stage.

Level	Taxon	Comparison	coef	qval
Class	Bacilli	SBM_F_vs_SBM_Mt	−4.0344	0.2185

A positive coefficient (coef) indicates that the former group in the comparison has a higher abundance, while a negative coefficient (coef) indicates the opposite. SBM = shoot-boring male, F = foregut, M1 = anterior midgut, M2 = posterior midgut, Mt = Malpighian tubules, H1 = ileum, H2 = colon, H3 = rectum.

To further validate the robustness of our primary findings, we performed a complementary non-parametric sensitivity analysis using Kruskal–Wallis tests followed by Dunn’s *post-hoc* comparisons with FDR correction on relative abundances. This sensitivity analysis was included because the Kruskal–Wallis test does not assume normality, making it particularly suitable for small sample sizes (*n* = 3 per group) and as a safeguard against potential outlier influences. The results from both methods were highly consistent for the key taxa reported, and the Kruskal–Wallis sensitivity analysis is presented in Supplementary Figure S6, S7.

All statistical analyses were performed in R (version 4.2.1) using the MaAsLin2 package (version 4.2.1) and the rstatix package for Kruskal–Wallis and Dunn tests.

#### 2.4.4 Definition of unique taxa

To standardize the identification of “unique” bacterial taxa across gut compartments, we applied the following criteria: an ASV was considered unique to a given compartment if it was detected with a prevalence ≥ 30% across biological replicates of that compartment and exhibited a mean relative abundance ≥ 0.1% within that compartment. ASVs that did not meet both thresholds were excluded from the Venn diagram and subsequent unique-taxon comparisons.

All graphical outputs (e.g., boxplots, ordination plots and heatmaps) were generated using the ggplot2 (v3.4.0) and pheatmap (v1.0.12) packages in R.

It should be noted that all alpha-diversity, beta-diversity, and differential abundance analyses in this study were based on relative abundances derived from 16S rRNA gene amplicon sequencing; absolute bacterial abundances were not measured. Therefore, our interpretations are limited to the relative abundance framework.

## 3 Results

### 3.1 Comparative analysis of bacterial communities in different gut compartments of *T. brevipilosus* across stages and sexes

Following aseptic dissection from the sampled specimens, the entire intestinal tract was separated into three anatomical regions-foregut, midgut, and hindgut-and the Malpighian tubules adherent to the intestinal wall were likewise isolated. Depending on whether fluorescence was observed, the midgut was further divided into two parts: the posterior portion was fluorescent, while the anterior portion was non-fluorescent. Additionally, based on anatomical differences, the hindgut was further partitioned into three distinct regions: the ileum, colon, and rectum (Rubio and Acuña, 2006; Candan et al., 2020; Koçakoğlu et al., 2020) (Figure 1A).

**Figure 1:**
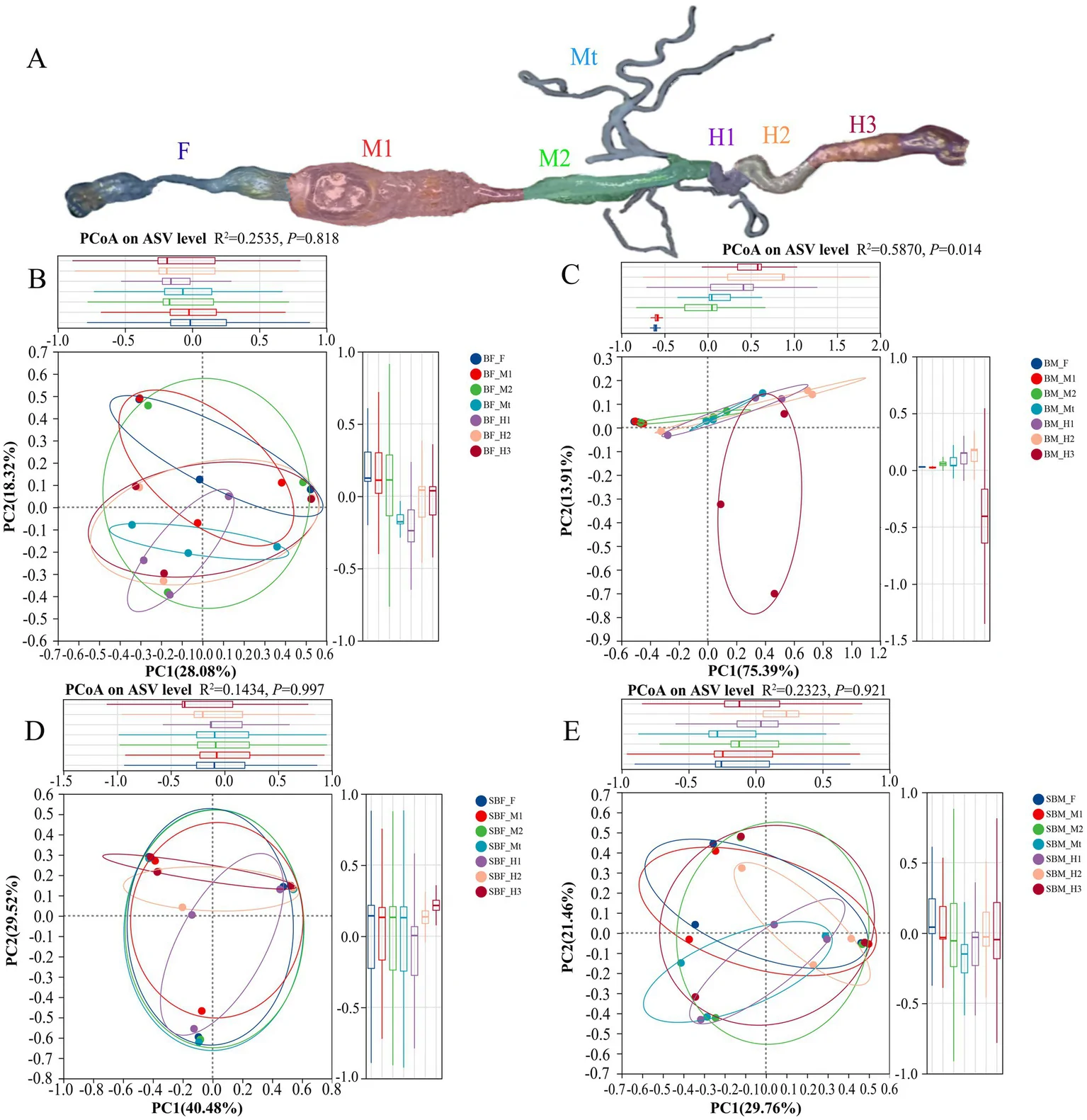
Differences in the composition of gut-associated bacterial communities among different gut compartments of male and female *Tomicus brevipilosus* adults during the trunk-boring and shoot-boring stages. **(A)** The adult gut was divided into six compartments and attached Malpighian tubules: the foregut (F), midgut (M1 and M2), Malpighian tubules (Mt), and hindgut (ileum: H1, colon: H2, and rectum: H3). **(B)** PCoA plot based on Bray-Curtis dissimilarities, showing gut-associated bacteria across seven compartments of trunk-boring phase female adults (PERMANOVA, *R*^2^ = 0.2535, *p* = 0.818, *n* = 3 per group). **(C)** PCoA plot based on Bray-Curtis dissimilarities, showing gut-associated bacteria across seven compartments of trunk-boring phase male adults (PERMANOVA, *R*^2^ = 0.5870, *p* = 0.014, *n* = 3 per group). **(D)** PCoA plot based on Bray-Curtis dissimilarities, showing gut-associated bacteria across seven compartments of female adults from shoot-boring phase (PERMANOVA, *R*^2^ = 0.1434, *p* = 0.997, *n* = 3 per group). **(E)** PCoA plot based on Bray-Curtis dissimilarities, showing gut-associated bacteria across seven compartments of male adults from shoot-boring phase (PERMANOVA, *R*^2^ = 0.2323, *p* = 0.921, *n* = 3 per group). Abbreviations for **(B,D)**: Foregut from female adults (F_F), midgut from female adults (F_M1 and F_M2), Malpighian tubules from female adults (F_Mt) and hindgut from female adults (F_H1, F_H2 and F_H3). Abbreviations for **(C,E)**: Foregut from male adults (M_F), midgut from male adults (M_M1 and M_M2), Malpighian tubules from male adults (M_Mt) and hindgut from male adults (M_H1, M_H2, and M_H3).

Based on ASV-level data, we first conducted PCoA analysis (Figures 1B–E). Each point in the plots corresponds to a replicate from different tissue samples, and the distances between samples reflect the degree of similarity in bacterial community composition and abundance-closer distances indicate higher similarity.

During the trunk-boring phase, global PERMANOVA (adonis2) tests revealed no significant overall differentiation among gut compartments in females (*R*^2^ = 0.2535, *p* = 0.818; Figure 1B). In males, however, a significant global difference was detected (*R*^2^ = 0.5870, *p* = 0.014; Figure 1C). Further pairwise comparisons showed that in males, the ileum (M_H1), colon (M_H2), rectum (M_H3), and Malpighian tubules (M_Mt) were significantly separated from the foregut (M_F) and anterior midgut (M_M1) (adjusted 𝑃 < 0.05 for all relevant contrasts; Supplementary Table S51), indicating pronounced intestinal compartmentalization. In contrast, during the shoot-boring phase, PERMANOVA (adonis2) tests detected no significant spatial segregation of gut compartments in either sex (females: *R*^2^ = 0.1434, *p* = 0.997; males: *R*^2^ = 0.2323, *p* = 0.921; Figures 1D,E).

These findings suggest that the trunk-boring period may impose greater selective pressure on compartmentalized bacterial communities compared to the shoot-boring stage.

### 3.2 Variations in gut bacterial alpha diversity among intestinal compartments of *T. brevipilosus*

During the trunk-boring phase, alpha-diversity analyses, performed using linear mixed-effects models (LMM) with Bonferroni post-hoc tests, revealed pronounced sex- and compartment-specific structuring of gut bacterial communities (Figure 2). In males, richness estimators (Sobs, Ace, and Chao) exhibited marked spatial heterogeneity along the gastrointestinal tract, with all three indices peaking in the rectum (*p* < 0.001 for the overall comparison across sections; Figures 2A–C and Supplementary Tables S1–S3). Pairwise comparisons showed that while the rectum and colon did not differ significantly from each other for any of these estimators, the rectum consistently outperformed all remaining compartments (foregut, anterior midgut, posterior midgut, ileum, and Malpighian tubules) (Figures 2A–C and Supplementary Tables S1–S3). This spatial gradient suggests that the male rectum harbors the most ASV-rich community, potentially reflecting its specialized role in digestive physiology. In stark contrast, females displayed a uniform richness pattern across all gut sections, with no significant inter-compartment differences (Figures 2A–C and Supplementary Tables S7–S9), indicating a more homogeneous distribution of bacterial diversity along the female intestinal tract.

**Figure 2:**
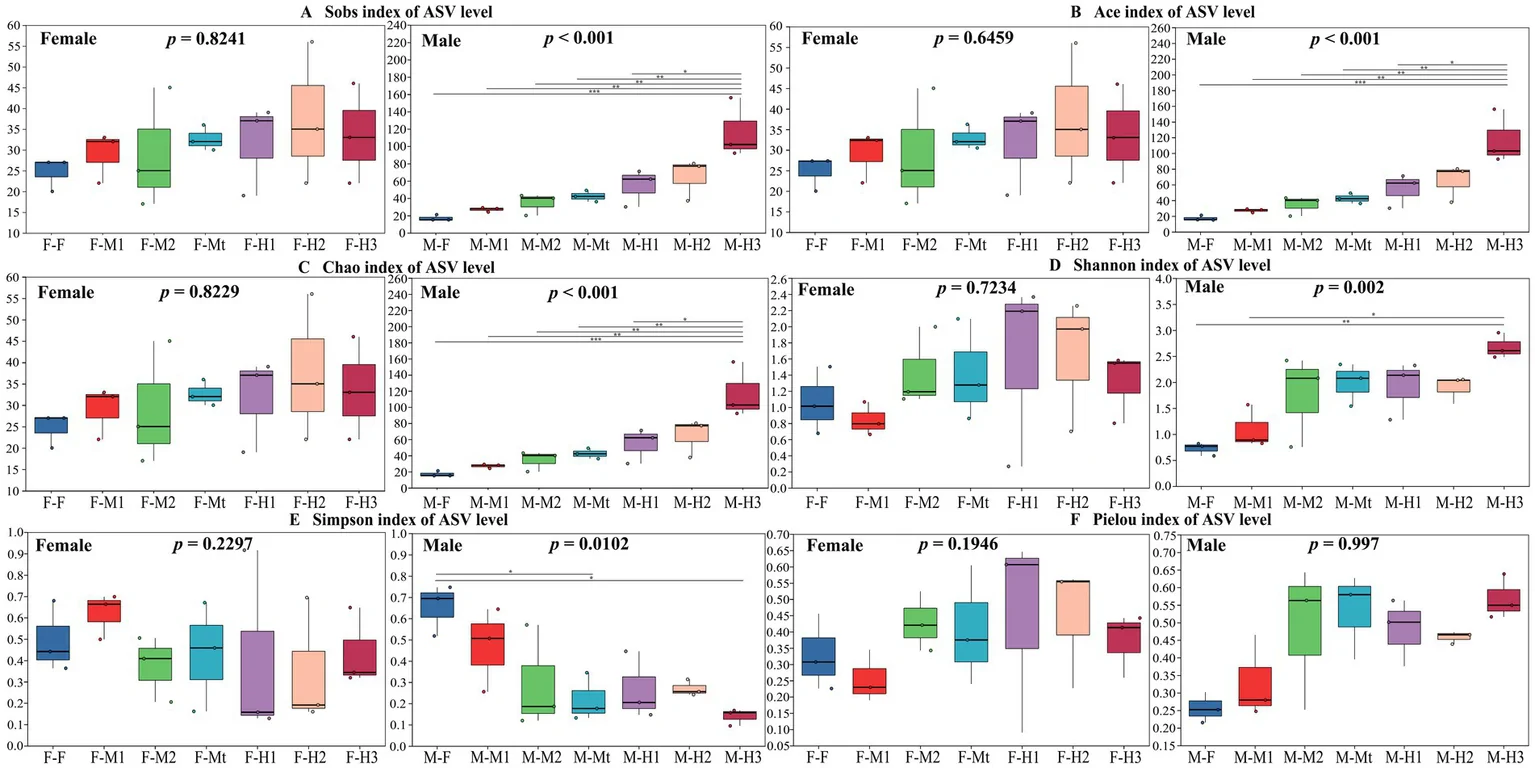
Alpha diversity of gut bacterial communities was assessed in the different intestinal compartments of both female and male *T. brevipilosus* adults from trunk-boring phase. Sobs **(A)**, Ace **(B)**, Chao **(C)**, Shannon **(D)**, Simpson **(E)**, and Pielou **(F)** were used to evaluate the bacterial diversity among different tissues. Abbreviations for **(A–F)**: Foregut from female adults (F-F), midgut from female adults (F-M1 and F-M2), Malpighian tubules from female adults (F-Mt), hindgut from female adults (F-H1, F-H2 and F-H3), foregut from male adults (M-F), midgut from male adults (M-M1 and M-M2), Malpighian tubules from male adults (M-Mt) and hindgut from male adults (M-H1, M-H2, and M-H3). All analyses are calculated on ASV levels and the presented values are the means ± standard deviation (SD). Linear mixed-effects models (LMMs) were fitted for each trait. Bonferroni-corrected post-hoc pairwise comparisons were conducted only when the omnibus *F*-test for the fixed effect of ‘compartment’ was globally significant (*p* < 0.05), indicating significant differences among compartments. Significance levels are denoted as: “*” for *p* < 0.05, “**” for *p* < 0.01 and “***” for *p* < 0.001.

The Shannon index, which integrates both richness and evenness, attained its maximum value in the male rectum, significantly exceeding values in the foregut and anterior midgut (Figure 2D and Supplementary Table S4). Conversely, the Simpson index-a measure of community dominance-was lowest in the same region, with significant differences relative to the foregut (Figure 2E and Supplementary Table S5). For female adults, no significant differences were observed across gut compartments for Shannon, Simpson, or Pielou indices (Figures 2D–F and Supplementary Tables S10–S12). Collectively, these patterns establish that during the trunk-boring stage, males exhibit spatially heterogeneous gut communities with progressively increasing diversity toward the hindgut, whereas females maintain spatially homogeneous communities with uniform diversity metrics throughout.

During the shoot-boring phase, the spatial patterns were markedly weaker. In females, the ileum displayed significantly higher Sobs compared to all other gut segments (Figure 3A and Supplementary Table S19); for the Chao index, the female ileum differed significantly from foregut (Figure 3C and Supplementary Table S21). No other alpha-diversity indices (Ace, Shannon, Simpson, Pielou) showed significant inter-compartment differences in either sex during this stage (Figures 3B,D–F and Supplementary Tables S19–S24 for females; Supplementary Tables S13–S18 for males). Thus, the shoot-boring phase is characterized by only limited spatial heterogeneity, with female ileum being the sole locus of elevated richness.

**Figure 3:**
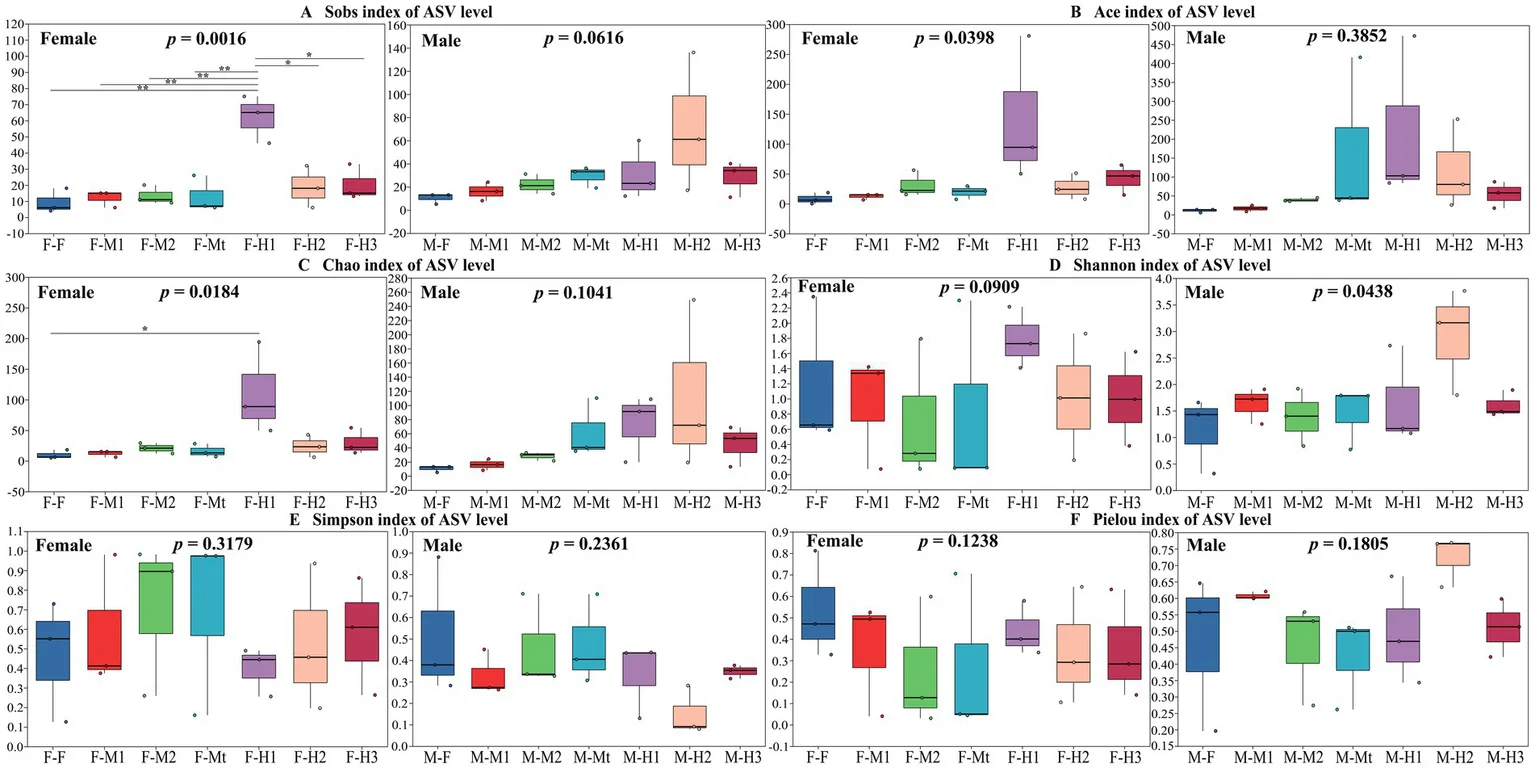
The alpha diversity of gut bacterial communities was assessed in the different intestinal compartments of both female and male *T. brevipilosus* adults from shoot-boring phase. Sobs **(A)**, Ace **(B)**, Chao **(C)**, Shannon **(D)**, Simpson **(E)**, and Pielou **(F)** were used to evaluate the bacterial diversity among different tissues. **(A–F)**: Foregut from female adults (F-F), midgut from female adults (F-M1 and F-M2), Malpighian tubules from female adults (F-Mt), hindgut from female adults (F-H1, F-H2 and F-H3), foregut from male adults (M-F), midgut from male adults (M-M1 and M-M2), Malpighian tubules from male adults (M-Mt) and hindgut from male adults (M-H1, M-H2 and M-H3). All analyses are calculated on ASV levels and the presented values are the means ± standard deviation (SD). Linear mixed-effects models (LMMs) were fitted for each trait. Bonferroni-corrected *post-hoc* pairwise comparisons were conducted only when the omnibus F-test for the fixed effect of ‘compartment’ was globally significant (*P* < 0.05), indicating significant differences among compartments. Significance levels are denoted as: “*” for *p* < 0.05, “**” for *p* < 0.01 and “***” for *p* < 0.001.

To formally assess the influence of sex and adult feeding phase on community assembly, we applied linear mixed-effects models (LMMs) coupled with Bonferroni post-hoc adjustments, which incorporated Sex × Compartment and Stage × Compartment interaction factors. The Sex × Compartment interaction was significant in both feeding stages, confirming that sexual differentiation of the gut microbiota is already established during the shoot-boring phase. In that stage, the interaction was significant for ASV richness (Sobs: *F* = 2.919, *p* = 0.0278) and Shannon diversity (*F* = 2.703, *p* = 0.0377). Pairwise contrasts further revealed that, within the colon, both Sobs (*F* = 16.497, *p* < 0.001) and Shannon (*F* = 9.089, *p* = 0.015) differed significantly between sexes, indicating that male–female divergence in gut compartmentalization is already detectable at this early life-history stage (Supplementary Figures S2A,D and Supplementary Tables S25, S28). Notably, the interaction intensified substantially during the trunk-boring phase, with highly significant effects across all three richness indices (Sobs: *F* = 7.957, *p* < 0.001; Ace: *F* = 7.039, *p* < 0.001; Chao: *F* = 8.069, *p* < 0.001). Importantly, sex-specific differences extended from the colon alone to both the colon and rectum for each richness metric: Sobs in colon (*F* = 7.255, *p* = 0.012) and rectum (*F* = 51.084, *p* < 0.001); Ace in colon (*F* = 4.800, *p* = 0.038) and rectum (*F* = 45.195, *p* < 0.001); and Chao in colon (*F* = 7.387, *p* = 0.011) and rectum (*F* = 51.796, *p* < 0.001) (Supplementary Figures S1A–C and Supplementary Tables S31–S33). The marked escalation in *F*-values (from ~2.7–2.9 to ~7.0–8.0) and the expansion of significantly affected richness metrics from two to three, together with the broadening of affected compartments from colon to both colon and rectum, collectively demonstrate that sexual dimorphism in gut bacterial community structure intensifies during the trunk-boring phase.

Analogously, the Stage × Compartment interaction was significant in both sexes, confirming that adult feeding phase also drives gut community differentiation. In females, the interaction was significant for Chao richness (*F* = 2.685, *p* = 0.0388) and Pielou evenness (*F* = 2.523, *p* = 0.0489). Pairwise comparisons indicated that Chao differed significantly between stages in the anterior midgut, Malpighian tubules, and ileum (*F* = 16.497, *p* < 0.001; Supplementary Figure S3C and Supplementary Tables S39, S46). In males, the interaction was more pronounced, with significant effects on Sobs (*F* = 3.859, *p* = 0.0078) and Shannon diversity (*F* = 3.432, *p* = 0.0137). Specifically, Shannon differed significantly between stages in both the colon (*F* = 4.515, *p* = 0.047) and rectum (*F* = 5.451, *p* = 0.031), while Sobs showed significant stage-related variation exclusively in the rectum (*F* = 24.566, *p* < 0.001) (Supplementary Figures S4A,D and Supplementary Tables S43, S46). These results indicate that the transition from shoot-boring to trunk-boring induces substantial microbial community reorganization, with the hindgut-particularly the rectum-being the primary site of temporal shifts, especially in males.

Our LMM-based analyses demonstrate that sexual dimorphism in the gut bacterial community of *T. brevipilosus* intensifies from the shoot-boring to the trunk-boring stage, with the Sex × Compartment interaction becoming markedly stronger and expanding from the colon to both the colon and rectum. During the trunk-boring phase, the male rectum emerges as a region of high ASV richness and diversity, exhibiting the highest richness, diversity, and evenness, whereas females display uniformly distributed communities across all gut compartments.

### 3.3 Bacterial community diversity in gut compartments of Adult *T. brevipilosus*

During the trunk-boring phase, male adults-particularly in the rectum-displayed markedly higher bacterial diversity than females or any other gut compartment, a trend consistent across all taxonomic levels. Overall, males harbored more taxa (e.g., 186 genera) than females (96 genera), with the male rectum alone accounting for 103 unique genera, the highest among all groups (Figures 4A,C,E,G,I; numerical details in legend). In the shoot-boring phase, both sexes showed elevated levels of unique taxa in the hindgut; however, the female ileum and male colon each exhibited the greatest richness of compartment-specific taxa (Figure 5; see legend for values).

**Figure 4:**
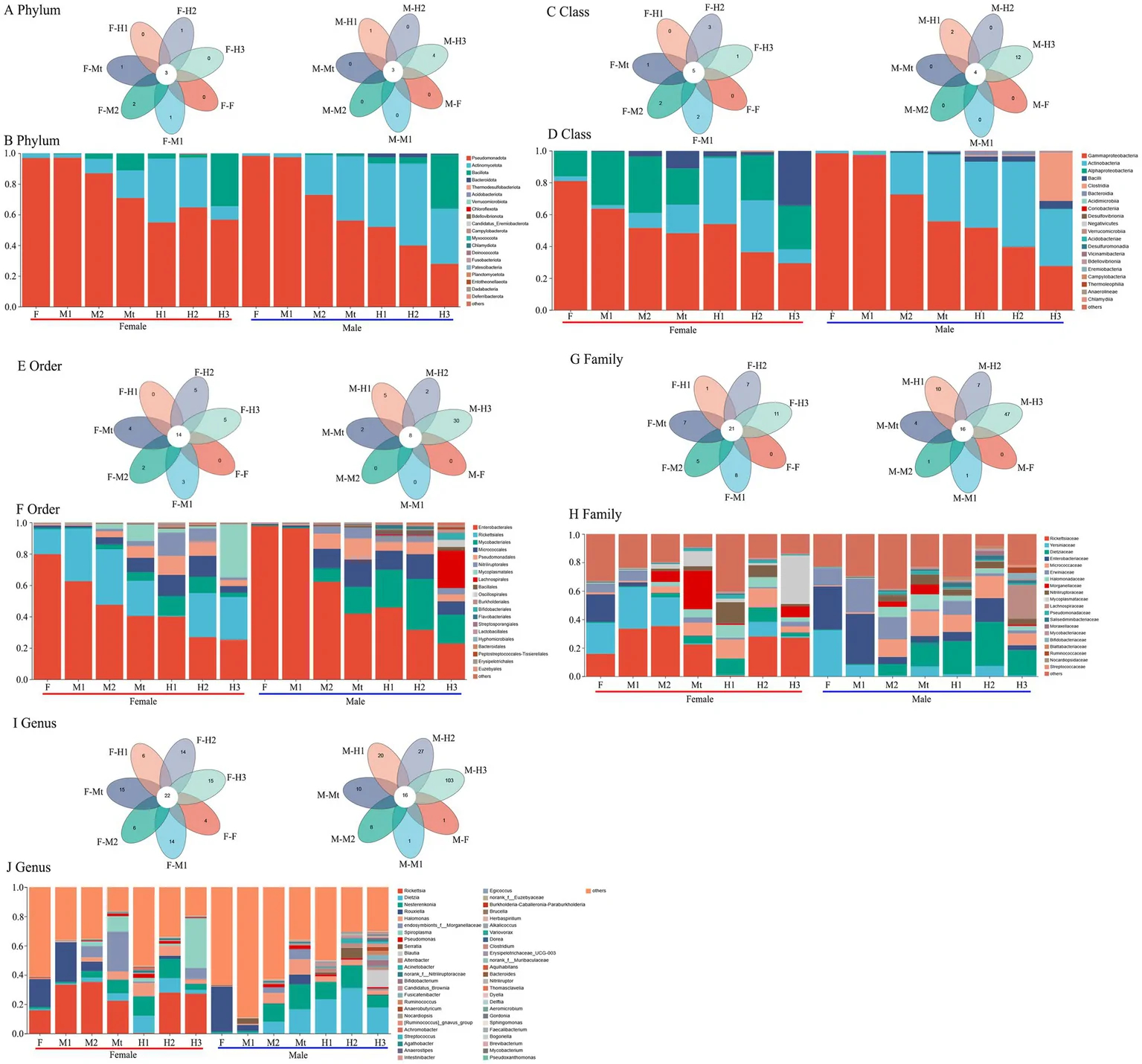
Relative abundance of gut bacterial phyla, classes, orders, families, genera in *T. brevipilosus* adults from trunk-boring phase. Distribution of bacterial community in *T. brevipilosus* at the phylum **(A,B)**, class **(C,D)**, order **(E,F)**, family **(G,H)**, and genus **(I,J)** levels. The numbers of phylum **(A)**, class **(C)**, order **(E)**, family **(G)** and genus **(I)** are shown by Venn diagrams. The relative abundance of bacteria are displayed at the phylum **(B)**, class **(D)**, order **(F)**, family **(H)** and genus **(J)** levels. Abbreviations for **(A,C,E,G,I)**: Foregut from female adults (F-F), midgut from female adults (F-M1 and F-M2), Malpighian tubules from female adults (F-Mt), hindgut from female adults (F-H1, F-H2 and F-H3), foregut from male adults (M-F), midgut from male adults (M-M1 and M-M2), Malpighian tubules from male adults (M-Mt) and hindgut from male adults (M-H1, M-H2 and M-H3). Abbreviations for **(B,D,F,H,J)**: Foregut from adults (F), midgut from adults (M1 and M2), Malpighian tubules from adults (Mt) and hindgut from adults (ileum: H1, colon: H2, and rectum: H3). Total bacterial taxa in males: 8 phyla, 18 classes, 47 orders, 86 families, 186 genera; in females: 8 phyla, 14 classes, 33 orders, 60 families, 96 genera. Unique taxa in the male rectum: 4 phyla, 12 classes, 30 orders, 47 families, 103 genera. Relative abundance rankings (descending order): Phylum: Pseudomonadota > Actinomycetota > Bacillota > Bacteroidota > Thermodesulfobacteriota > Acidobacteriota > Verrucomicrobiota; Class: Gammaproteobacteria > Actinobacteria > Alphaproteobacteria > Bacilli > Clostridia > Bacteroidia > Acidimicrobiia > Coriobacteriia > Desulfovibrionia > Negativicutes > Verrucomicrobiia > Acidobacteriae > Desulfuromonadia; Order: Enterobacterales > Rickettsiales > Mycobacteriales > Micrococcales > Pseudomonadales > Nitriliruptorales > Mycoplasmatales > Lachnospirales > Bacillales > Oscillospirales; Family: Rickettsiaceae > Yersiniaceae > Dietziaceae > Enterobacteriaceae > Micrococcaceae > Erwiniaceae > Halomonadaceae > Morganellaceae > Nitriliruptoraceae > Mycoplasmataceae > Lachnospiraceae > Pseudomonadaceae > Salisediminibacteriaceae > Moraxellaceae; Genus: *Rickettsia* > *Dietzia* > *Nesterenkonia* > *Rouxiella* > *Halomonas* > *Spiroplasma* > *Pseudomonas* > *Serratia* > *Blautia* > *Alteribacter* > *Acinetobacter*.

**Figure 5:**
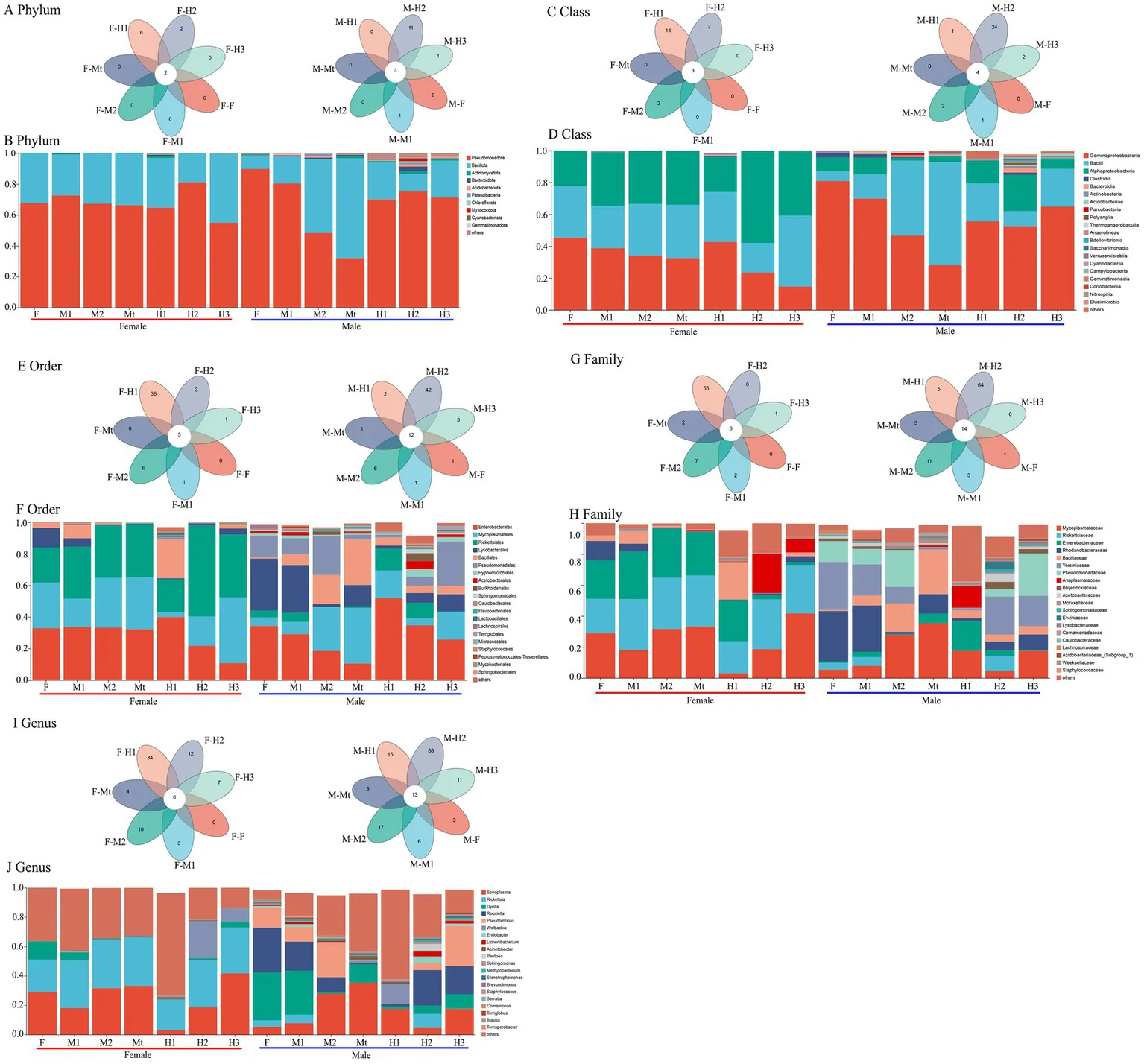
Relative abundance of gut bacterial phyla, classes, orders, families, genera in *T. brevipilosus* adults from shoot-boring phase. Distribution of bacterial community in *T. brevipilosus* at the phylum **(A,B)**, class **(C,D)**, order **(E,F)**, family **(G,H)**, and genus **(I,J)** levels. The numbers of phylum **(A)**, class **(C)**, order **(E)**, family **(G)** and genus **(I)** are shown by Venn diagrams. Frequencies of bacteria are displayed at the phylum **(B)**, class **(D)**, order **(F)**, family **(H)** and genus **(J)** levels. Abbreviations for **(A,C,E,G,I)**: Foregut from female adults (F-F), midgut from female adults (F-M1 and F-M2), Malpighian tubules from female adults (F-Mt), hindgut from female adults (F-H1, F-H2 and F-H3), foregut from male adults (M-F), midgut from male adults (M-M1 and M-M2), Malpighian tubules from male adults (M-Mt) and hindgut from male adults (M-H1, M-H2 and M-H3). Abbreviations for **(B,D,F,H,J)**: Foregut from adults (F), midgut from adults (M1 and M2), Malpighian tubules from adults (Mt) and hindgut from adults (H1, H2 and H3). Unique taxa in the female ileum: 6 phyla, 14 classes, 36 orders, 55 families, 84 genera; in the male colon: 11 phyla, 24 classes, 42 orders, 64 families, 88 genera. Relative abundance rankings were broadly similar to those observed in the trunk-boring phase.

With respect to relative abundance, the ranking of dominant lineages was similar across hierarchical levels during the trunk-boring phase: at the phylum level, Pseudomonadota was most abundant, followed by Actinomycetota, Bacillota, etc.; at the genus level, *Rickettsia* predominated (Figures 4B,D,F,H,J; full rankings in legend). Stacked bar plots further revealed that the rectal community structure of males diverged distinctly from other intestinal regions. Venn diagrams corroborated this, showing that the male rectum contained substantially more unique taxa at every taxonomic level than any other sample (Figure 4). In the shoot-boring phase, sex-related differences were less pronounced, yet the female ileum and male colon consistently harbored significantly more unique taxa than other segments of the same sex (Figure 5).

### 3.4 Stage- and sex-specific Bacteria in the intestinal compartments of *T. brevipilosus*

MaAsLin2 analysis was used to analyze the relative abundances from phylum to genus, with significance set at FDR-adjusted *q* < 0.25. In trunk-boring males, the hindgut-especially segment rectum-emerged as the most prominent enrichment site for multiple bacterial lineages. At the phylum level, Bacillota and Actinomycetota were consistently more abundant in rectum compared to several other segments (e.g., vs. M1 and F; Table 2). This enrichment was hierarchically supported within Bacillota: the classes Bacilli and Clostridia, and their descendant orders-including Bacillales, Lactobacillales, Lachnospirales, Oscillospirales, Peptostreptococcales-Tissierellales, and Clostridiales-all showed elevated abundances in rectum. At the family level, Lachnospiraceae, Ruminococcaceae, and Oscillospiraceae followed the same trend. Within Actinomycetota, the class Actinobacteria and a broad set of orders-Mycobacteriales, Micrococcales, Streptosporangiales, Propionibacteriales, Coriobacteriales, and Bifidobacteriales-were significantly higher in rectum. This cascade continued to families such as Dietziaceae, Mycobacteriaceae, Micrococcaceae, and Nocardiopsidaceae, and finally to the genus *Dietzia*, which was enriched in rectum (Table 2 and Supplementary Figure S6F). Additionally, the genus *Acinetobacter* (phylum Pseudomonadota, class Gammaproteobacteria, order Pseudomonadales, family Moraxellaceae) was also significantly more abundant in rectum, despite no overall phylum-level difference (Table 2). Other enriched classes included Acidimicrobiia, Bacteroidia, and Coriobacteriia, which were higher in rectum versus selected segments. The genera *Lactobacillus*, *Alteribacter*, *Nesterenkonia*, and *Nocardiopsis* showed significant enrichment in the ileum (Table 2).

Trunk-boring females showed fewer significant hindgut differences (Table 1). Within Bacillota, the order Bacillales and Lachnospirales were lower in foregut than in rectum (Table 1). In Pseudomonadota, the genus *Delftia* (class Betaproteobacteria) was enriched in rectum (Table 1). *Halomonas* and *Alteribacter* were also enriched in rectum (Table 1).

In the shoot-boring stage, females exhibited hindgut subregion heterogeneity (Table 3). The phylum Actinomycetota and its class Actinobacteria were significantly elevated in ileum relative to colon and marginally versus rectum (Table 3). The class Clostridia (phylum Bacillota) was lower in foregut than in ileum and higher in ileum than in Malpighian tubule, rectum, and anterior of midgut (Table 3). The phylum Patescibacteria and its class Parcubacteria were depleted in foregut compared to ileum (Table 3). At the genus level, female adults did not exhibit any particularly distinct or specific bacterial genera (Table 3). Shoot-boring males had no gut-related significant associations (Table 4).

### 3.5 Functional metabolic potential of bacteria in different intestinal compartments of *Tomicus brevipilosus* adults across different stages and sexes

Using PICRUSt2-based functional prediction, we identified positive potential associations during the trunk-boring phase between hindgut-specific bacteria and multiple KEGG pathways in both females and males. These included pathways involved in metabolism of cofactors and vitamins, energy metabolism, membrane transport, amino acid metabolism, and carbohydrate metabolism (Figures 6A,B) and the predicted functional profiles for the shoot-boring phase are presented in Figure 7.

**Figure 6:**
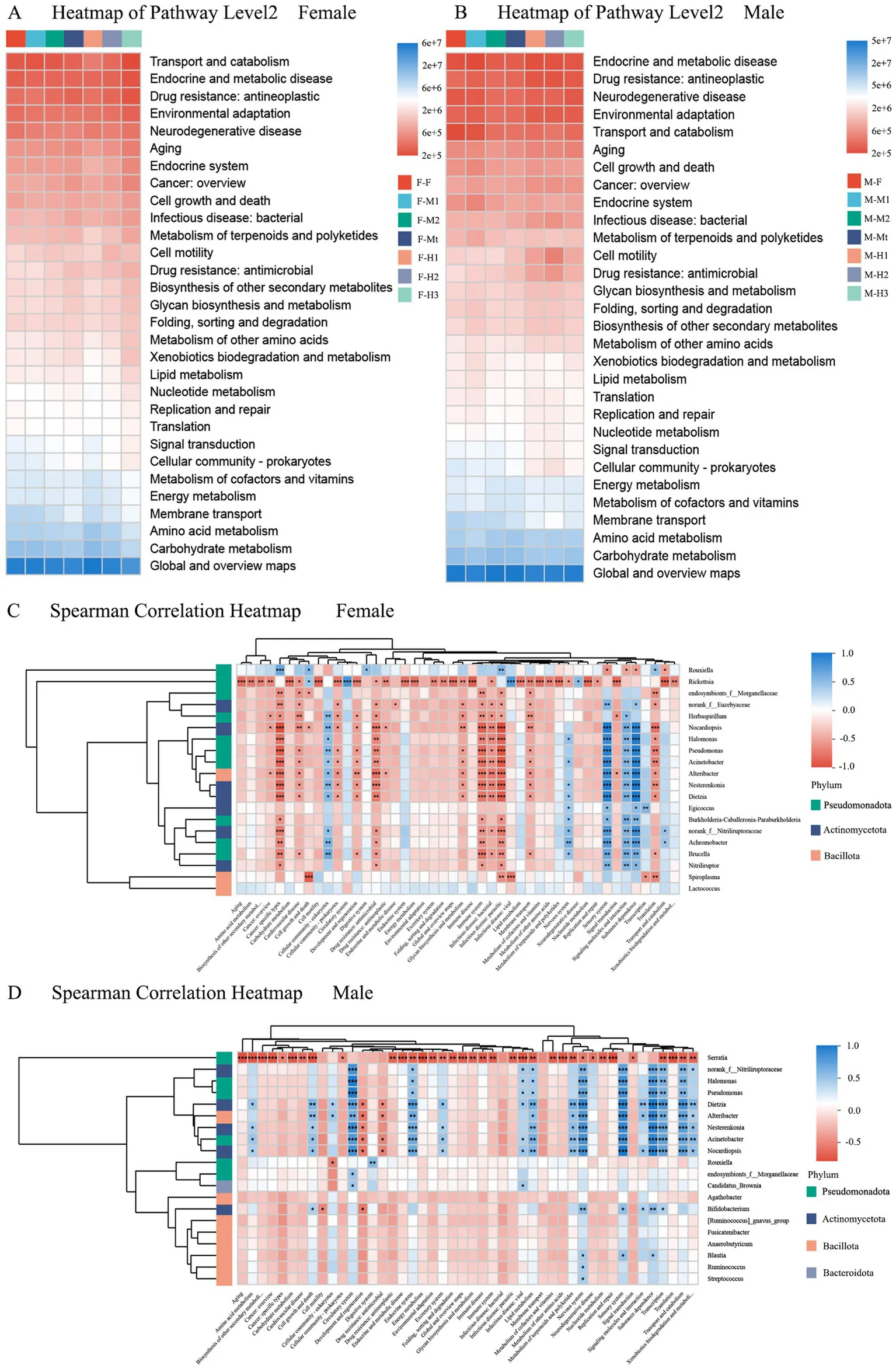
Functional potential prediction (PICRUSt2) of the 20 most abundant gut bacterial taxa in adult females and males of *T. brevipilosus* from trunk-boring phase. Thermal representation of microbiota-associated KEGG pathways (Level 2) across different intestinal compartments in female **(A)** and male **(B)** adults during the trunk-boring phase. Spearman correlation heatmap between KEGG pathways (Level 2) and associated gut bacteria in female **(C)** and male **(D)** adults across various intestinal compartments throughout the same period. To improve readability, the textual content of figures that were hard to decipher **(C,D)** has been reproduced in Supplementary material 2.

**Figure 7:**
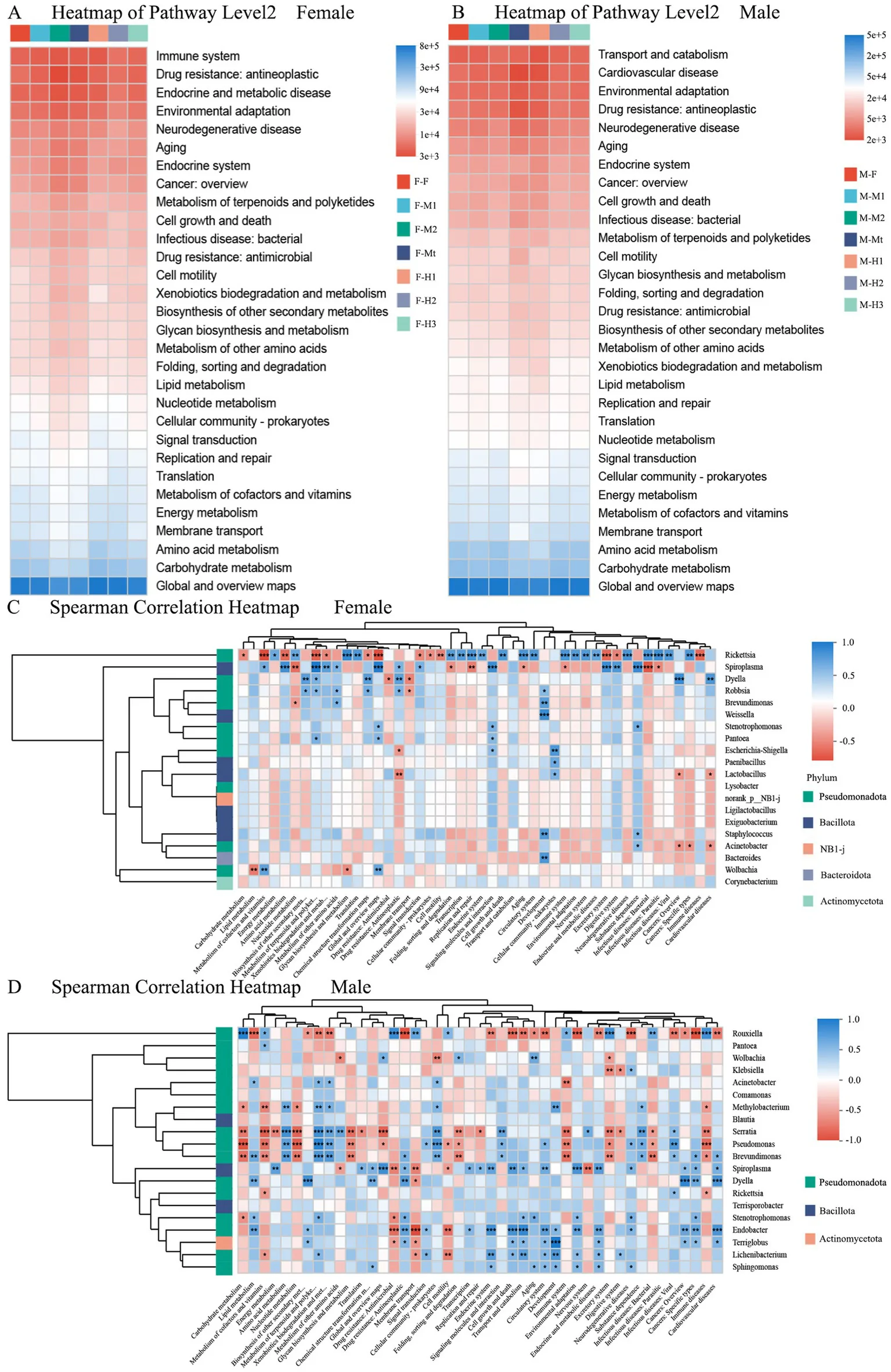
Functional potential prediction (PICRUSt2) of the 20 most abundant gut bacterial taxa in adult females and males of *T. brevipilosus* from shoot-boring phase. Thermal representation of microbiota-associated KEGG pathways (Level 2) across different intestinal compartments in female **(A)** and male **(B)** adults during the shoot-boring phase. Spearman correlation heatmap between KEGG pathways (Level 2) and associated gut bacteria in female **(C)** and male **(D)** adults across various gut compartments throughout the same period. To improve readability, the textual content of figures that were hard to decipher **(C,D)** has been reproduced in Supplementary material 2.

Our findings revealed that during the trunk-boring stage, the gut bacterial communities of adult males and females exhibited marked differences in both compositional and functional profiles, and these features were closely associated with host physiology. In males, *Alteribacter*, *Nesterenkonia*, and *Nocardiopsis* were significantly enriched in the ileum (Table 2). These genera showed broad positive potential correlations with diverse metabolic and systems-level pathways-including amino acid metabolism, lipid metabolism, and signal transduction-while displaying negative potential associations with antimicrobial resistance pathways. In addition, the male rectum harbored enriched populations of *Dietzia* and *Acinetobacter*, which were primarily potentially correlated with amino acid and lipid metabolism (Figure 6D). In contrast, in females, *Delftia*, *Halomonas*, and *Alteribacter* were enriched in the rectum (Table 1), and these genera exhibited positive potential correlations with signaling interaction-related pathways, but negative potential correlations with antimicrobial resistance pathways (Figure 6C).

## 4 Discussion

Based on morphological landmarks, the gut of *T. brevipilosus* was divided into six distinct gut compartments plus the attached Malpighian tubules. At the late shoot-boring stage, *T. brevipilosus* shifts from feeding on shoots to boring into the trunk phloem, where it constructs an oviposition chamber. Subsequently, females undertake oviposition, whereas males assume the role of clearing excreta and dietary debris from the galleries with their abdominal apex and elytral declivity. Niche shifts in phytophagous insects are inextricably intertwined with their gut microbiota (Zhang et al., 2023). Concurrently, the transition to trunk-boring results in varying degrees of divergence in the gut compartment bacteria between male and female adults. Previous studies have shown that wood-boring insects often exhibit pronounced gut compartmentalization. For instance, each gut compartment of the wood-feeding beetle *Odontotaenius disjunctus* harbors a unique microbial community (Ceja-Navarro et al., 2019). In this study, we observed that during the trunk-boring stage, male beetles exhibited more pronounced changes in their gut compartment bacteria than females. Within males, the rectum emerged as a compartment of high ASV richness and diversity, characterized by the highest ASV richness, evenness, and community complexity. The rectum of males during the trunk-boring stage was enriched with several unique bacterial genera, all of which were short-chain fatty acid producers. We tentatively hypothesize that the short-chain fatty acids produced by these bacteria might function to inhibit harmful microbes within the galleries and brood chambers (Lehenberger et al., 2025). This study additionally revealed that *Delftia* and *Halomonas*, both enriched in the female gut, are capable of cellulose degradation, potentially providing essential nutrients to support female reproductive activities. This pattern may constitute an adaptation driven by the sex-specific behavioral divergence of adults observed during the trunk-boring period. Similarly, the gut bacterial community of the phloem-feeding bark beetle *Dendroctonus armandi* also differs between sexes (Hu et al., 2013).

In *T. brevipilosus*, the gut together with its associated Malpighian tubules comprised four main parts: the foregut, midgut, Malpighian tubules, and hindgut. The hindgut itself was further partitioned into the ileum, colon, and rectum (Rubio and Acuña, 2006; Candan et al., 2020; Koçakoğlu et al., 2020). Autofluorescence was observed in the posterior portion of the midgut but not in the anterior portion; based on this differential distribution, the midgut was separated into two distinct regions-an anterior midgut and a posterior midgut (Figure 1A). During the shoot-boring stage, no notable regional differentiation in gut bacterial communities was observed in either sex (Figures 1D,E). In the trunk-boring phase, marked compartment-specific differentiation was observed in males, but was not evident in females (Figures 1B,C and Supplementary Table S51). The results suggest that the transition to trunk-boring promotes marked differentiation of gut bacterial assemblages across distinct intestinal compartments of *T. brevipilosus*. This finding was consistent with previous studies: in the rotten-wood-feeding beetle *Pelidnota luridipes* (Falqueto et al., 2022), and the phloem-feeding longhorn beetle *Apriona swainsoni* (Zhang et al., 2025), the hindgut was the gut region with the highest bacterial ASV richness, diversity, or enrichment. These convergent patterns across disparate insect lineages suggest that differentiation of gut bacteria across distinct intestinal compartments may represent a conserved adaptive strategy in herbivorous insects during the trunk-boring period.

In the trunk-boring stage, the male rectum stood out as a region of high ASV richness and diversity, exhibiting the highest values across multiple diversity metrics-including ASV richness, community complexity, ASV evenness, and high equitability (Figure 2 and Supplementary Tables S1–S6). In contrast, females displayed spatially homogeneous communities, with similar diversity metrics across all gut compartments (Figure 2 and Supplementary Tables S7–S12). We used LMMs with Sex × Compartment and Stage × Compartment interactions (Bonferroni). The Sex interaction was significant during shoot-boring stage and intensified in trunk-boring stage, expanding to colon + rectum for all richness indices-demonstrating stronger sexual dimorphism with stage (Supplementary Tables S25–S36). The Stage interaction was significant in both sexes: females showed shifts in anterior midgut, Malpighian tubules, and ileum (Chao, evenness), whereas males showed pronounced changes in colon and rectum (Sobs, Shannon), especially in rectum (Supplementary Tables S37–S48). For instance, in the phloem-feeding bark beetle *Dendroctonus armandi*, the gut bacterial community also differs between males and females (Hu et al., 2013).

At various taxonomic levels, bar charts (Figures 4B,D,F,H,J) and Venn diagram analyses (Figures 4A,C,E,G,I) consistently showed that in trunk-boring males, the rectum from hindgut harbored a distinct bacterial community composition and contained significantly more unique taxa than all other intestinal segments. MaAsLin2 results revealed that the male rectum served as a region of elevated ASV richness and diversity, with 2 phyla, 6 classes, 12 orders, 7 families, and 2 genera (namely *Dietzia* and *Acinetobacter*) showing significantly higher abundances in this gut compartment (Table 2 and Supplementary Figure S6F). Both genera are known SCFA producers: *Dietzia* produces acetic acid (Rani et al., 2022), while *Acinetobacter* can also generate SCFAs (Guo et al., 2021).

Short-chain fatty acid exert numerous beneficial effects on insects (Zheng et al., 2017; Liang et al., 2026). They alleviate intestinal damage in *Zophobas morio* and protect the gut barrier by inhibiting intestinal pathogens and increasing intestinal immune factors (Liang et al., 2026). SCFAs also promote the growth of honeybee (Zheng et al., 2017). In *Anastrepha ludens*, the absence of acetic acid bacteria in the gut impairs development on *Psidium guajava* (Ochoa-Sánchez et al., 2022). Butyrate extends the lifespan of *Apis mellifera* and improves learning and memory abilities impaired by D-galactose (Zhou et al., 2025). Volatile acetic acid released by four *Enterococcus* strains promotes oviposition in *Sitophilus zeamais* (Beato et al., 2025). Acetic acid in the gut of *Zootermopsis nevadensis* exerts a significant inhibitory effect on *Serratia marcescens*, a bacterium with pathogenic potential (Inagaki and Matsuura, 2018).

The female adult of *T. brevipilosus* bores into the phloem of *P. yunnanensis* to construct an egg gallery, where it releases aggregation pheromones to attract males. Upon arrival, the male does not mate immediately but first produces courtship acoustic signals (Chen et al., 2015). Only after the female receives and recognizes these signals does it exit the gallery to mate. Following copulation, the female re-enters the gallery to lay eggs, while the male assists by removing debris and frass from the gallery (Chen et al., 2015). Fungi introduced into the galleries by *T. brevipilosus* proliferate extensively (Wang et al., 2019), which may affect beetle development. If the above hypothesis holds, male adults could potentially suppress the proliferation of such microorganisms in the galleries through rectal excretion of feces that contain SCFAs or SCFA-producing bacteria. The antimicrobial properties of SCFAs are well documented. For instance, organic acids produced by probiotics, such as acetic and propionic acid, reduce the viability of pathogenic microorganisms (Zavisic et al., 2024). Butyrate exhibits antimicrobial activity against a broad range of fungi and bacteria (Zutz et al., 2017) and can protect wood from degradation by pathogenic fungi, including *Phanerochaete chrysosporium*, *Trametes menziesii*, and white-rot species (Hussain et al., 2024). Additionally, acetic acid produced by Enterococcus completely inhibits the growth of Fusarium verticillioides at a concentration of 1 mM (Gergolet Diaz et al., 2021), whereas acetic acid from the wood-colonizing fungus *A. hylecoeti* suppresses competing fungi while promoting its own growth (Lehenberger et al., 2025). Collectively, these findings are consistent with the scenario that SCFAs in male frass might serve a protective role against microbial pathogens within the gallery, although this correlative inference requires direct experimental validation.

In contrast, the bacterial communities across the gut segments of females were more uniformly distributed. Notably, *Delftia* and *Halomonas*, which were enriched in the rectum (Table 2), are both capable of cellulose degradation (Narayanan et al., 2025; Wang et al., 2025), potentially providing sufficient nutrients to support female reproductive activities. However, this interpretation remains correlative and requires functional validation through culture-based experiments and gene expression analyses.

During the shoot-boring stage, no significant differentiation of bacterial communities was observed among the gut compartments in either male or female beetles (Figures 1D,E). At this stage, female (Table 3) and male (Table 4) adults did not exhibit any particularly distinct or specific bacterial genera.

The pronounced sexual dimorphism in gut bacterial architecture observed during the trunk-boring stage likely reflects divergent behavioral and physiological demands between males and females. Males are engaged in gallery maintenance and fecal removal, and the short-chain fatty acids (SCFAs) produced by distinct bacterial taxa enriched in their rectums may hypothetically inhibit the growth of pathogenic microbes within the galleries. In females, gut bacteria might participate in cellulose degradation, thereby supporting reproductive activities. In contrast, during the shoot-boring stage, the bacterial communities across different gut segments of both sexes clustered together, and neither female nor male adults exhibited any particularly distinct or specific bacterial genera. This pattern suggests an adaptive response of the gut microbiota to the host’s niche shift.

We acknowledge that the relatively small sample size-only three biological replicates per group (each comprising 20 pooled individuals)-constitutes a limitation of this study. Nevertheless, the extremely low *p*-values and large effect sizes observed for key comparisons-including alpha-diversity indices, *β*-diversity differentiation, and differential abundance of specific taxa-strongly indicate that the detected patterns are highly robust and unlikely to be due to small sample size. Moreover, the functional predictions reported herein are derived from 16S rRNA gene sequencing data and thus represent inferred metabolic potential rather than direct metabolic activity. Future work employing orthogonal approaches-such as metabolomics, transcriptomics, or cultivation-based experiments-will be necessary to substantiate these predictions. Additionally, it should be acknowledged that our microbial analyses were confined to the gut compartments of *T. brevipilosus* adults, and we did not characterize the bacterial communities associated with the shoot or phloem tissues of *Pinus yunnanensis* on which the beetles feed. Given that herbivorous insects can acquire or be influenced by diet-associated microorganisms, the absence of plant tissue microbiome data limits our ability to discern whether the observed stage- and sex-specific gut community shifts are primarily driven by direct dietary acquisition, by host physiological reprogramming during the niche transition, or by their interplay. Future studies that simultaneously profile the microbiomes of both the insect gut and the ingested plant tissues will be valuable for disentangling these potential mechanisms.

Future research will focus on elucidating the functions of sex-specific bacteria in male and female beetles, and on leveraging these host-beneficial bacteria to manage and control this devastating forest pest.

## 5 Conclusion

In summary, this study revealed that during the shoot-boring stage, when *T. brevipilosus* fed on shoots, no significant differentiation of bacterial communities was observed among gut compartments in either sex. No distinct genus-level specificity was observed at this stage. During the trunk-boring stage, *T. brevipilosus* adults exhibit clear sex-specific roles: females are engaged in oviposition, whereas the males employ their abdominal apex and elytral declivity to expel waste from the tunnels. At this stage, the gut bacterial communities display pronounced compartmental and sex-specific differentiation. Male recta exhibited the highest levels of bacterial ASV richness, evenness, and community complexity, and harbored two SCFA-producing bacteria specific to this region. We speculate that the SCFAs produced by these bacteria may enhance host defense against pathogens encountered within tunnels. Conversely, female adults showed no obvious spatial partitioning of gut bacteria, although *Delftia* and *Halomonas* were preferentially enriched in the rectum, where they might contribute to cellulose breakdown and thus facilitate reproduction. These functional inferences, however, await direct experimental confirmation.

## Data Availability

The datasets presented in this study can be found in online repositories. The names of the repository/repositories and accession number(s) can be found at: https://www.ncbi.nlm.nih.gov/, PRJNA1451412.

## Footnotes

1
https://cloud.majorbio.com

## Supplementary material

SUPPLEMENTARY FIGURE S1 Linear mixed‑effects models (LMMs) were used to assess the sex‑by‑compartment interaction on alpha diversity indices-Sobs **(A)**, Ace **(B)**, Chao **(C)**, Shannon **(D)**, Simpson **(E)**, and Pielou **(F)** across the seven gut segments during the trunk‑boring stage. To formally test whether compartmentalization differs between males and females, separate LMMs were fitted with 'sex', 'compartment', and their interaction as fixed factors. Post‑hoc pairwise comparisons, adjusted with Bonferroni correction, were performed to dissect the interaction for each sex‑specific contrast. Significance levels are denoted as: “*” for *p* < 0.05, “**” for *p* < 0.01 and “***” for *p* < 0.001.

SUPPLEMENTARY FIGURE S2 Linear mixed‑effects models (LMMs) were used to assess the sex‑by‑compartment interaction on alpha diversity indices-Sobs **(A)**, Ace **(B)**, Chao **(C)**, Shannon **(D)**, Simpson **(E)**, and Pielou **(F)** across the seven gut segments during the shoot‑boring stage. To formally test whether compartmentalization differs between males and females, separate LMMs were fitted with 'sex', 'compartment', and their interaction as fixed factors. *Post‑hoc* pairwise comparisons, adjusted with Bonferroni correction, were performed to dissect the interaction for each sex‑specific contrast. Significance levels are denoted as: “*” for *p* < 0.05, “**” for *p* < 0.01 and “***” for *p* < 0.001.

SUPPLEMENTARY FIGURE S3 Linear mixed‑effects models (LMMs) were used to assess the stage‑by‑compartment interaction on alpha diversity indices-Sobs **(A)**, Ace **(B)**, Chao **(C)**, Shannon **(D)**, Simpson **(E)**, and Pielou **(F)** across the seven gut segments in female individuals. To formally test whether compartmentalization differs between the trunk‑boring and shoot‑boring stages, we fitted separate LMMs with 'stage', 'compartment', and their interaction as fixed factors. Post‑hoc pairwise comparisons, adjusted using the Bonferroni correction, were performed to dissect the interaction for each stage‑specific contrast. Significance levels are denoted as: “*” for *p* < 0.05, “**” for *p* < 0.01 and “***” for *p* < 0.001.

SUPPLEMENTARY FIGURE S4 Linear mixed‑effects models (LMMs) were used to assess the stage‑by‑compartment interaction on alpha diversity indices-Sobs **(A)**, Ace **(B)**, Chao **(C)**, Shannon **(D)**, Simpson **(E)**, and Pielou **(F)** across the seven gut segments in male individuals. To formally test whether compartmentalization differs between the trunk‑boring and shoot‑boring stages, we fitted separate LMMs with 'stage', 'compartment', and their interaction as fixed factors. *Post‑hoc* pairwise comparisons, adjusted using the Bonferroni correction, were performed to dissect the interaction for each stage‑specific contrast. Significance levels are denoted as: “*” for *p* < 0.05, “**” for *p* < 0.01 and “***” for *p* < 0.001.

SUPPLEMENTARY FIGURE S5 PCR Electrophoresis Banding Patterns of Bacterial Communities in Different Intestinal Segments of Male and Female Adults During the Trunk-Boring and Shoot-Boring Stages. In A, lanes 1–42 correspond to the following samples from the trunk-boring stage: F_F_1, F_F_2, F_F_3, F_M1_1, F_M1_2, F_M1_3, F_M2_1, F_M2_2, F_M2_3, F_Mt_1, F_Mt_2, F_Mt_3, F_H1_1, F_H1_2, F_H1_3, F_H2_1, F_H2_2, F_H2_3, F_H3_1, F_H3_2, F_H3_3, M_F_1, M_F_2, M_F_3, M_M1_1, M_M1_2, M_M1_3, M_M2_1, M_M2_2, M_M2_3, M_Mt_1, M_Mt_2, M_Mt_3, M_H1_1, M_H1_2, M_H1_3, M_H2_1, M_H2_2, M_H2_3, M_H3_1, M_H3_2, and M_H3_3. In B, lanes 1-42 represent the corresponding samples from the shoot-boring stage, with the same labeling scheme. Lane M denotes the standard DNA marker, and lane CK indicates the negative control, which consisted of a mixture (prepared at equal volume ratios) of all extraction kit reagents (buffers and solutions) and nuclease‑free water used in the bacterial 16S PCR amplification; this mixture was then subjected to PCR and electrophoresis under identical conditions. For the shoot‑boring stage, samples F_H2_3 (lane 15), M_H1_2 (lane 32), and M_Mt_2 (lane 41) did not yield discernible bands in the initial extraction; therefore, a second extraction was performed using the remaining pooled insect specimens, which successfully produced clear bands. Similarly, sample M_H1_1 (lane 31) from the shoot‑boring stage showed no detectable bands after either the first or second extraction; consequently, a third extraction was carried out on the collected specimens, which ultimately resulted in distinct bands.

SUPPLEMENTARY FIGURE S6 Comparison of the abundances of specific bacteria in different intestinal compartments of male and female *Tomicus brevipilosus* adults during the trunk‑boring stage, at the order, family, and genus levels. The distribution proportions of the most abundant order **(A,B)**, family **(C,D),** and genus **(E,F)** are compared using Kruskal–Wallis *H* test. Abbreviations for **(A–F)**: foregut (F-F), midgut (F M1, F-M2), Malpighian tubules (F-Mt), and hindgut (F-H1, F-H2, F-H3) from female adults; and foregut (M-F), midgut (M-M1, M-M2), Malpighian tubules (M-Mt), and hindgut (M-H1, M-H2, M H3) from male adults. Significance levels are denoted as: “*” for *p* < 0.05, “**” for *p* < 0.01 and “***” for *p* < 0.001.

SUPPLEMENTARY FIGURE S7 Comparison of the abundances of specific bacteria in different intestinal compartments of male and female *Tomicus brevipilosus* adults during the shoot‑boring stage, at the phylum, class, order, family, and genus levels. The distribution proportions of the most abundant phylum **(A,B)**, class **(C,D)**, order **(E,F)**, family **(G,H)** and genus **(I,J)** are compared using Kruskal–Wallis *H* test. Abbreviations for **(A–J)**: foregut (F-F), midgut (F-M1, F-M2), Malpighian tubules (F-Mt), and hindgut (F-H1, F-H2, F-H3) from female adults; and foregut (M-F), midgut (M-M1, M-M2), Malpighian tubules (M-Mt), and hindgut (M-H1, M-H2, M-H3) from male adults. Significance levels are denoted as: “*” for *p* < 0.05, “**” for *p* < 0.01 and “***” for *p* < 0.001.
