# Supplementary material for: Bacterial diversity in gut compartments of Tomicus brevipilosus: from shoot-boring preconditioning of Pinus yunnanensis to trunk-boring reproduction
Source: Front Microbiol. 2026 Oct 2;17:1914356. doi: 10.3389/fmicb.2026.1914356 (PMC13672192; doi:10.3389/fmicb.2026.1914356)
Supplement: Supplementary file 1 [file Table_1.DOCX]

**Supplementary Material**

**Supplementary Tables**

Supplementary Table S1. Results of linear mixed-effects models (LMMs) evaluating the variation in the Sobs index (alpha diversity) among gut compartments of male adults during the trunk-boring stage. To identify specific compartmental differences, post-hoc pairwise comparisons were conducted, and the family-wise error rate was controlled by applying the Bonferroni adjustment to all derived *P*-values

| **Sobs** | ***F* = 9.747** | ***p*_value = 4.978825195138759E-4** |
| --- | --- | --- |
|  |  | *p*_value |
| F vs. | M1 | 1 |
|  | M2 | 1 |
|  | Mt | 1 |
|  | H1 | 0.602 |
|  | H2 | 0.168 |
|  | H3 | 5.055173129846032E-4 |
| M1 vs. | F | 1 |
|  | M2 | 1 |
|  | Mt | 1 |
|  | H1 | 1 |
|  | H2 | 0.578 |
|  | H3 | 0.0013659429264569287 |
| M2 vs. | F | 1 |
|  | M1 | 1 |
|  | Mt | 1 |
|  | H1 | 1 |
|  | H2 | 1 |
|  | H3 | 0.003 |
| Mt vs. | F | 1 |
|  | M1 | 1 |
|  | M2 | 1 |
|  | H1 | 1 |
|  | H2 | 1 |
|  | H3 | 0.007 |
| H1 vs. | F | 0.602 |
|  | M1 | 1 |
|  | M2 | 1 |
|  | Mt | 1 |
|  | H2 | 1 |
|  | H3 | 0.028 |
| H2 vs. | F | 0.168 |
|  | M1 | 0.578 |
|  | M2 | 1 |
|  | Mt | 1 |
|  | H1 | 1 |
|  | H3 | 0.099 |
| H3 vs. | F | 5.055173129846032E-4 |
|  | M1 | 0.0013659429264569287 |
|  | M2 | 0.003 |
|  | Mt | 0.007 |
|  | H1 | 0.028 |
|  | H2 | 0.099 |

Supplementary Table S2. Results of linear mixed-effects models (LMMs) evaluating the variation in the Ace index (alpha diversity) among gut compartments of male adults during the trunk-boring stage. To identify specific compartmental differences, post-hoc pairwise comparisons were conducted, and the family-wise error rate was controlled by applying the Bonferroni adjustment to all derived *P*-values

| **Ace** | ***F* = 9.999** | ***p*_value = 4.4128810494947133E-4** |
| --- | --- | --- |
|  |  | *p*_value |
| F vs. | M1 | 1 |
|  | M2 | 1 |
|  | Mt | 1 |
|  | H1 | 0.585 |
|  | H2 | 0.158 |
|  | H3 | 0 |
| M1 vs. | F | 1 |
|  | M2 | 1 |
|  | Mt | 1 |
|  | H1 | 1 |
|  | H2 | 0.548 |
|  | H3 | 0.0012143512858268045 |
| M2 vs. | F | 1 |
|  | M1 | 1 |
|  | Mt | 1 |
|  | H1 | 1 |
|  | H2 | 1 |
|  | H3 | 0.003 |
| Mt vs. | F | 1 |
|  | M1 | 1 |
|  | M2 | 1 |
|  | H1 | 1 |
|  | H2 | 1 |
|  | H3 | 0.006 |
| H1 vs. | F | 0.585 |
|  | M1 | 1 |
|  | M2 | 1 |
|  | Mt | 1 |
|  | H2 | 1 |
|  | H3 | 0.025 |
| H2 vs. | F | 0.158 |
|  | M1 | 0.548 |
|  | M2 | 1 |
|  | Mt | 1 |
|  | H1 | 1 |
|  | H3 | 0.09 |
| H3 vs. | F | 0 |
|  | M1 | 0.0012143512858268045 |
|  | M2 | 0.003 |
|  | Mt | 0.006 |
|  | H1 | 0.025 |
|  | H2 | 0.09 |

Supplementary Table S3. Results of linear mixed-effects models (LMMs) evaluating the variation in the Chao index (alpha diversity) among gut compartments of male adults during the trunk-boring stage. To identify specific compartmental differences, post-hoc pairwise comparisons were conducted, and the family-wise error rate was controlled by applying the Bonferroni adjustment to all derived *P*-values

| **Chao** | ***F* = 9.911** | ***p*_value = 4.6018357450847207E-4** |
| --- | --- | --- |
|  |  | *p*_value |
| F vs. | M1 | 1 |
|  | M2 | 1 |
|  | Mt | 1 |
|  | H1 | 0.587 |
|  | H2 | 0.16 |
|  | H3 | 0 |
| M1 vs. | F | 1 |
|  | M2 | 1 |
|  | Mt | 1 |
|  | H1 | 1 |
|  | H2 | 0.552 |
|  | H3 | 0.001265244861845374 |
| M2 vs. | F | 1 |
|  | M1 | 1 |
|  | Mt | 1 |
|  | H1 | 1 |
|  | H2 | 1 |
|  | H3 | 0.003 |
| Mt vs. | F | 1 |
|  | M1 | 1 |
|  | M2 | 1 |
|  | H1 | 1 |
|  | H2 | 1 |
|  | H3 | 0.007 |
| H1 vs. | F | 0.587 |
|  | M1 | 1 |
|  | M2 | 1 |
|  | Mt | 1 |
|  | H2 | 1 |
|  | H3 | 0.026 |
| H2 vs. | F | 0.16 |
|  | M1 | 0.552 |
|  | M2 | 1 |
|  | Mt | 1 |
|  | H1 | 1 |
|  | H3 | 0.094 |
| H3 vs. | F | 0 |
|  | M1 | 0.001265244861845374 |
|  | M2 | 0.003 |
|  | Mt | 0.007 |
|  | H1 | 0.026 |
|  | H2 | 0.094 |

Supplementary Table S4. Results of linear mixed-effects models (LMMs) evaluating the variation in the Shannon index (alpha diversity) among gut compartments of male adults during the trunk-boring stage. To identify specific compartmental differences, post-hoc pairwise comparisons were conducted, and the family-wise error rate was controlled by applying the Bonferroni adjustment to all derived *P*-values

| **Shannon** | ***F* = 7.171** | ***p*_value = 0.00199363975161455** |
| --- | --- | --- |
|  |  | *p*_value |
| F vs. | M1 | 1 |
|  | M2 | 0.215 |
|  | Mt | 0.059 |
|  | H1 | 0.088 |
|  | H2 | 0.098 |
|  | H3 | 0.002 |
| M1 vs. | F | 1 |
|  | M2 | 1 |
|  | Mt | 0.447 |
|  | H1 | 0.669 |
|  | H2 | 0.747 |
|  | H3 | 0.011 |
| M2 vs. | F | 0.215 |
|  | M1 | 1 |
|  | Mt | 1 |
|  | H1 | 1 |
|  | H2 | 1 |
|  | H3 | 0.362 |
| Mt vs. | F | 0.059 |
|  | M1 | 0.447 |
|  | M2 | 1 |
|  | H1 | 1 |
|  | H2 | 1 |
|  | H3 | 1 |
| H1 vs. | F | 0.088 |
|  | M1 | 0.669 |
|  | M2 | 1 |
|  | Mt | 1 |
|  | H2 | 1 |
|  | H3 | 0.878 |
| H2 vs. | F | 0.098 |
|  | M1 | 0.747 |
|  | M2 | 1 |
|  | Mt | 1 |
|  | H1 | 1 |
|  | H3 | 0.787 |
| H3 vs. | F | 0.002 |
|  | M1 | 0.011 |
|  | M2 | 0.362 |
|  | Mt | 1 |
|  | H1 | 0.878 |
|  | H2 | 0.787 |

Supplementary Table S5. Results of linear mixed-effects models (LMMs) evaluating the variation in the Simpson index (alpha diversity) among gut compartments of male adults during the trunk-boring stage. To identify specific compartmental differences, post-hoc pairwise comparisons were conducted, and the family-wise error rate was controlled by applying the Bonferroni adjustment to all derived *P*-values

| **Simpson** | ***F* = 4.793** | ***p*_value = 0.010216302730193114** |
| --- | --- | --- |
|  |  | *p*_value |
| F vs. | M1 | 1 |
|  | M2 | 0.156 |
|  | Mt | 0.047 |
|  | H1 | 0.102 |
|  | H2 | 0.11 |
|  | H3 | 0.013 |
| M1 vs. | F | 1 |
|  | M2 | 1 |
|  | Mt | 0.957 |
|  | H1 | 1 |
|  | H2 | 1 |
|  | H3 | 0.265 |
| M2 vs. | F | 0.156 |
|  | M1 | 1 |
|  | Mt | 1 |
|  | H1 | 1 |
|  | H2 | 1 |
|  | H3 | 1 |
| Mt vs. | F | 0.047 |
|  | M1 | 0.957 |
|  | M2 | 1 |
|  | H1 | 1 |
|  | H2 | 1 |
|  | H3 | 1 |
| H1 vs. | F | 0.102 |
|  | M1 | 1 |
|  | M2 | 1 |
|  | Mt | 1 |
|  | H2 | 1 |
|  | H3 | 1 |
| H2 vs. | F | 0.11 |
|  | M1 | 1 |
|  | M2 | 1 |
|  | Mt | 1 |
|  | H1 | 1 |
|  | H3 | 1 |
| H3 vs. | F | 0.013 |
|  | M1 | 0.265 |
|  | M2 | 1 |
|  | Mt | 1 |
|  | H1 | 1 |
|  | H2 | 1 |

Supplementary Table S6. Results of linear mixed-effects models (LMMs) evaluating the variation in the Pielou index (alpha diversity) among gut compartments of male adults during the trunk-boring stage. To identify specific compartmental differences, post-hoc pairwise comparisons were conducted, and the family-wise error rate was controlled by applying the Bonferroni adjustment to all derived *P*-values

| **Pielou** | ***F* = 0.082** | ***p*_value = 0.9970474747766404** |
| --- | --- | --- |
|  |  | *p*_value |
| F vs. | M1 | 1 |
|  | M2 | 1 |
|  | Mt | 1 |
|  | H1 | 1 |
|  | H2 | 1 |
|  | H3 | 1 |
| M1 vs. | F | 1 |
|  | M2 | 1 |
|  | Mt | 1 |
|  | H1 | 1 |
|  | H2 | 1 |
|  | H3 | 1 |
| M2 vs. | F | 1 |
|  | M1 | 1 |
|  | Mt | 1 |
|  | H1 | 1 |
|  | H2 | 1 |
|  | H3 | 1 |
| Mt vs. | F | 1 |
|  | M1 | 1 |
|  | M2 | 1 |
|  | H1 | 1 |
|  | H2 | 1 |
|  | H3 | 1 |
| H1 vs. | F | 1 |
|  | M1 | 1 |
|  | M2 | 1 |
|  | Mt | 1 |
|  | H2 | 1 |
|  | H3 | 1 |
| H2 vs. | F | 1 |
|  | M1 | 1 |
|  | M2 | 1 |
|  | Mt | 1 |
|  | H1 | 1 |
|  | H3 | 1 |
| H3 vs. | F | 1 |
|  | M1 | 1 |
|  | M2 | 1 |
|  | Mt | 1 |
|  | H1 | 1 |
|  | H2 | 1 |

Supplementary Table S7. Results of linear mixed-effects models (LMMs) evaluating the variation in the Sobs index (alpha diversity) among gut compartments of female adults during the trunk-boring stage. To identify specific compartmental differences, post-hoc pairwise comparisons were conducted, and the family-wise error rate was controlled by applying the Bonferroni adjustment to all derived *P*-values

| **Sobs** | ***F* = 0.461** | ***p*_value = 0.8241141093681055** |
| --- | --- | --- |
|  |  | *p*_value |
| F vs. | M1 | 1 |
|  | M2 | 1 |
|  | Mt | 1 |
|  | H1 | 1 |
|  | H2 | 1 |
|  | H3 | 1 |
| M1 vs. | F | 1 |
|  | M2 | 1 |
|  | Mt | 1 |
|  | H1 | 1 |
|  | H2 | 1 |
|  | H3 | 1 |
| M2 vs. | F | 1 |
|  | M1 | 1 |
|  | Mt | 1 |
|  | H1 | 1 |
|  | H2 | 1 |
|  | H3 | 1 |
| Mt vs. | F | 1 |
|  | M1 | 1 |
|  | M2 | 1 |
|  | H1 | 1 |
|  | H2 | 1 |
|  | H3 | 1 |
| H1 vs. | F | 1 |
|  | M1 | 1 |
|  | M2 | 1 |
|  | Mt | 1 |
|  | H2 | 1 |
|  | H3 | 1 |
| H2 vs. | F | 1 |
|  | M1 | 1 |
|  | M2 | 1 |
|  | Mt | 1 |
|  | H1 | 1 |
|  | H3 | 1 |
| H3 vs. | F | 1 |
|  | M1 | 1 |
|  | M2 | 1 |
|  | Mt | 1 |
|  | H1 | 1 |
|  | H2 | 1 |

Supplementary Table S8. Results of linear mixed-effects models (LMMs) evaluating the variation in the Ace index (alpha diversity) among gut compartments of female adults during the trunk-boring stage. To identify specific compartmental differences, post-hoc pairwise comparisons were conducted, and the family-wise error rate was controlled by applying the Bonferroni adjustment to all derived *P*-values

| **Ace** | ***F* = 0.714** | ***p*_value = 0.6459102722235579** |
| --- | --- | --- |
|  |  | *p*_value |
| F vs. | M1 | 1 |
|  | M2 | 1 |
|  | Mt | 1 |
|  | H1 | 1 |
|  | H2 | 1 |
|  | H3 | 1 |
| M1 vs. | F | 1 |
|  | M2 | 1 |
|  | Mt | 1 |
|  | H1 | 1 |
|  | H2 | 1 |
|  | H3 | 1 |
| M2 vs. | F | 1 |
|  | M1 | 1 |
|  | Mt | 1 |
|  | H1 | 1 |
|  | H2 | 1 |
|  | H3 | 1 |
| Mt vs. | F | 1 |
|  | M1 | 1 |
|  | M2 | 1 |
|  | H1 | 1 |
|  | H2 | 1 |
|  | H3 | 1 |
| H1 vs. | F | 1 |
|  | M1 | 1 |
|  | M2 | 1 |
|  | Mt | 1 |
|  | H2 | 1 |
|  | H3 | 1 |
| H2 vs. | F | 1 |
|  | M1 | 1 |
|  | M2 | 1 |
|  | Mt | 1 |
|  | H1 | 1 |
|  | H3 | 1 |
| H3 vs. | F | 1 |
|  | M1 | 1 |
|  | M2 | 1 |
|  | Mt | 1 |
|  | H1 | 1 |
|  | H2 | 1 |

Supplementary Table S9. Results of linear mixed-effects models (LMMs) evaluating the variation in the Chao index (alpha diversity) among gut compartments of female adults during the trunk-boring stage. To identify specific compartmental differences, post-hoc pairwise comparisons were conducted, and the family-wise error rate was controlled by applying the Bonferroni adjustment to all derived *P*-values

| **Chao** | ***F* = 0.463** | ***p*_value = 0.8229064008961627** |
| --- | --- | --- |
|  |  | *p*_value |
| F vs. | M1 | 1 |
|  | M2 | 1 |
|  | Mt | 1 |
|  | H1 | 1 |
|  | H2 | 1 |
|  | H3 | 1 |
| M1 vs. | F | 1 |
|  | M2 | 1 |
|  | Mt | 1 |
|  | H1 | 1 |
|  | H2 | 1 |
|  | H3 | 1 |
| M2 vs. | F | 1 |
|  | M1 | 1 |
|  | Mt | 1 |
|  | H1 | 1 |
|  | H2 | 1 |
|  | H3 | 1 |
| Mt vs. | F | 1 |
|  | M1 | 1 |
|  | M2 | 1 |
|  | H1 | 1 |
|  | H2 | 1 |
|  | H3 | 1 |
| H1 vs. | F | 1 |
|  | M1 | 1 |
|  | M2 | 1 |
|  | Mt | 1 |
|  | H2 | 1 |
|  | H3 | 1 |
| H2 vs. | F | 1 |
|  | M1 | 1 |
|  | M2 | 1 |
|  | Mt | 1 |
|  | H1 | 1 |
|  | H3 | 1 |
| H3 vs. | F | 1 |
|  | M1 | 1 |
|  | M2 | 1 |
|  | Mt | 1 |
|  | H1 | 1 |
|  | H2 | 1 |

Supplementary Table S10. Results of linear mixed-effects models (LMMs) evaluating the variation in the Shannon index (alpha diversity) among gut compartments of female adults during the trunk-boring stage. To identify specific compartmental differences, post-hoc pairwise comparisons were conducted, and the family-wise error rate was controlled by applying the Bonferroni adjustment to all derived *P*-values

| **Shannon** | ***F* = 0.603** | ***p*_value = 0.7233553212227475** |
| --- | --- | --- |
|  |  | *p*_value |
| F vs. | M1 | 1 |
|  | M2 | 1 |
|  | Mt | 1 |
|  | H1 | 1 |
|  | H2 | 1 |
|  | H3 | 1 |
| M1 vs. | F | 1 |
|  | M2 | 1 |
|  | Mt | 1 |
|  | H1 | 1 |
|  | H2 | 1 |
|  | H3 | 1 |
| M2 vs. | F | 1 |
|  | M1 | 1 |
|  | Mt | 1 |
|  | H1 | 1 |
|  | H2 | 1 |
|  | H3 | 1 |
| Mt vs. | F | 1 |
|  | M1 | 1 |
|  | M2 | 1 |
|  | H1 | 1 |
|  | H2 | 1 |
|  | H3 | 1 |
| H1 vs. | F | 1 |
|  | M1 | 1 |
|  | M2 | 1 |
|  | Mt | 1 |
|  | H2 | 1 |
|  | H3 | 1 |
| H2 vs. | F | 1 |
|  | M1 | 1 |
|  | M2 | 1 |
|  | Mt | 1 |
|  | H1 | 1 |
|  | H3 | 1 |
| H3 vs. | F | 1 |
|  | M1 | 1 |
|  | M2 | 1 |
|  | Mt | 1 |
|  | H1 | 1 |
|  | H2 | 1 |

Supplementary Table S11. Results of linear mixed-effects models (LMMs) evaluating the variation in the Simpson index (alpha diversity) among gut compartments of female adults during the trunk-boring stage. To identify specific compartmental differences, post-hoc pairwise comparisons were conducted, and the family-wise error rate was controlled by applying the Bonferroni adjustment to all derived *P*-values

| **Simpson** | ***F* = 1.6** | ***p*_value = 0.22967388491415888** |
| --- | --- | --- |
|  |  | *p*_value |
| F vs. | M1 | 1 |
|  | M2 | 1 |
|  | Mt | 1 |
|  | H1 | 1 |
|  | H2 | 1 |
|  | H3 | 1 |
| M1 vs. | F | 1 |
|  | M2 | 1 |
|  | Mt | 1 |
|  | H1 | 1 |
|  | H2 | 0.398 |
|  | H3 | 1 |
| M2 vs. | F | 1 |
|  | M1 | 1 |
|  | Mt | 1 |
|  | H1 | 1 |
|  | H2 | 1 |
|  | H3 | 1 |
| Mt vs. | F | 1 |
|  | M1 | 1 |
|  | M2 | 1 |
|  | H1 | 1 |
|  | H2 | 1 |
|  | H3 | 1 |
| H1 vs. | F | 1 |
|  | M1 | 1 |
|  | M2 | 1 |
|  | Mt | 1 |
|  | H2 | 0.622 |
|  | H3 | 1 |
| H2 vs. | F | 1 |
|  | M1 | 0.398 |
|  | M2 | 1 |
|  | Mt | 1 |
|  | H1 | 0.622 |
|  | H3 | 1 |
| H3 vs. | F | 1 |
|  | M1 | 1 |
|  | M2 | 1 |
|  | Mt | 1 |
|  | H1 | 1 |
|  | H2 | 1 |

Supplementary Table S12. Results of linear mixed-effects models (LMMs) evaluating the variation in the Pielou index (alpha diversity) among gut compartments of female adults during the trunk-boring stage. To identify specific compartmental differences, post-hoc pairwise comparisons were conducted, and the family-wise error rate was controlled by applying the Bonferroni adjustment to all derived *P*-values

| **Pielou** | ***F* = 1.741** | ***p*_value = 0.1946406132950465** |
| --- | --- | --- |
|  |  | *p*_value |
| F vs. | M1 | 1 |
|  | M2 | 1 |
|  | Mt | 1 |
|  | H1 | 1 |
|  | H2 | 0.951 |
|  | H3 | 1 |
| M1 vs. | F | 1 |
|  | M2 | 1 |
|  | Mt | 1 |
|  | H1 | 1 |
|  | H2 | 0.269 |
|  | H3 | 1 |
| M2 vs. | F | 1 |
|  | M1 | 1 |
|  | Mt | 1 |
|  | H1 | 1 |
|  | H2 | 1 |
|  | H3 | 1 |
| Mt vs. | F | 1 |
|  | M1 | 1 |
|  | M2 | 1 |
|  | H1 | 1 |
|  | H2 | 1 |
|  | H3 | 1 |
| H1 vs. | F | 1 |
|  | M1 | 1 |
|  | M2 | 1 |
|  | Mt | 1 |
|  | H2 | 0.825 |
|  | H3 | 1 |
| H2 vs. | F | 0.951 |
|  | M1 | 0.269 |
|  | M2 | 1 |
|  | Mt | 1 |
|  | H1 | 0.825 |
|  | H3 | 1 |
| H3 vs. | F | 1 |
|  | M1 | 1 |
|  | M2 | 1 |
|  | Mt | 1 |
|  | H1 | 1 |
|  | H2 | 1 |

Supplementary Table S13. Results of linear mixed-effects models (LMMs) evaluating the variation in the Sobs index (alpha diversity) among gut compartments of male adults during the shoot-boring stage. To identify specific compartmental differences, post-hoc pairwise comparisons were conducted, and the family-wise error rate was controlled by applying the Bonferroni adjustment to all derived *P*-values

| **Sobs** | ***F* = 2.79** | ***p*_value = 0.06159838688678281** |
| --- | --- | --- |
|  |  | *p*_value |
| F vs. | M1 | 1 |
|  | M2 | 1 |
|  | Mt | 1 |
|  | H1 | 1 |
|  | H2 | 0.076 |
|  | H3 | 1 |
| M1 vs. | F | 1 |
|  | M2 | 1 |
|  | Mt | 1 |
|  | H1 | 1 |
|  | H2 | 0.15 |
|  | H3 | 1 |
| M2 vs. | F | 1 |
|  | M1 | 1 |
|  | Mt | 1 |
|  | H1 | 1 |
|  | H2 | 0.234 |
|  | H3 | 1 |
| Mt vs. | F | 1 |
|  | M1 | 1 |
|  | M2 | 1 |
|  | H1 | 1 |
|  | H2 | 0.388 |
|  | H3 | 1 |
| H1 vs. | F | 1 |
|  | M1 | 1 |
|  | M2 | 1 |
|  | Mt | 1 |
|  | H2 | 0.582 |
|  | H3 | 1 |
| H2 vs. | F | 0.076 |
|  | M1 | 0.15 |
|  | M2 | 0.234 |
|  | Mt | 0.388 |
|  | H1 | 0.582 |
|  | H3 | 0.339 |
| H3 vs. | F | 1 |
|  | M1 | 1 |
|  | M2 | 1 |
|  | Mt | 1 |
|  | H1 | 1 |
|  | H2 | 0.339 |

Supplementary Table S14. Results of linear mixed-effects models (LMMs) evaluating the variation in the Ace index (alpha diversity) among gut compartments of male adults during the shoot-boring stage. To identify specific compartmental differences, post-hoc pairwise comparisons were conducted, and the family-wise error rate was controlled by applying the Bonferroni adjustment to all derived *P*-values

| **Ace** | ***F* = 1.15** | ***p*_value = 0.38523346335932057** |
| --- | --- | --- |
|  |  | *p*_value |
| F vs. | M1 | 1 |
|  | M2 | 1 |
|  | Mt | 1 |
|  | H1 | 1 |
|  | H2 | 1 |
|  | H3 | 1 |
| M1 vs. | F | 1 |
|  | M2 | 1 |
|  | Mt | 1 |
|  | H1 | 1 |
|  | H2 | 1 |
|  | H3 | 1 |
| M2 vs. | F | 1 |
|  | M1 | 1 |
|  | Mt | 1 |
|  | H1 | 1 |
|  | H2 | 1 |
|  | H3 | 1 |
| Mt vs. | F | 1 |
|  | M1 | 1 |
|  | M2 | 1 |
|  | H1 | 1 |
|  | H2 | 1 |
|  | H3 | 1 |
| H1 vs. | F | 1 |
|  | M1 | 1 |
|  | M2 | 1 |
|  | Mt | 1 |
|  | H2 | 1 |
|  | H3 | 1 |
| H2 vs. | F | 1 |
|  | M1 | 1 |
|  | M2 | 1 |
|  | Mt | 1 |
|  | H1 | 1 |
|  | H3 | 1 |
| H3 vs. | F | 1 |
|  | M1 | 1 |
|  | M2 | 1 |
|  | Mt | 1 |
|  | H1 | 1 |
|  | H2 | 1 |

Supplementary Table S15. Results of linear mixed-effects models (LMMs) evaluating the variation in the Chao index (alpha diversity) among gut compartments of male adults during the shoot-boring stage. To identify specific compartmental differences, post-hoc pairwise comparisons were conducted, and the family-wise error rate was controlled by applying the Bonferroni adjustment to all derived *P*-values

| **Chao** | ***F* = 2.294** | ***p*_value = 0.10411181386333773** |
| --- | --- | --- |
|  |  | *p*_value |
| F vs. | M1 | 1 |
|  | M2 | 1 |
|  | Mt | 1 |
|  | H1 | 1 |
|  | H2 | 0.175 |
|  | H3 | 1 |
| M1 vs. | F | 1 |
|  | M2 | 1 |
|  | Mt | 1 |
|  | H1 | 1 |
|  | H2 | 0.258 |
|  | H3 | 1 |
| M2 vs. | F | 1 |
|  | M1 | 1 |
|  | Mt | 1 |
|  | H1 | 1 |
|  | H2 | 0.545 |
|  | H3 | 1 |
| Mt vs. | F | 1 |
|  | M1 | 1 |
|  | M2 | 1 |
|  | H1 | 1 |
|  | H2 | 0.818 |
|  | H3 | 1 |
| H1 vs. | F | 1 |
|  | M1 | 1 |
|  | M2 | 1 |
|  | Mt | 1 |
|  | H2 | 1 |
|  | H3 | 1 |
| H2 vs. | F | 0.175 |
|  | M1 | 0.258 |
|  | M2 | 0.545 |
|  | Mt | 0.818 |
|  | H1 | 1 |
|  | H3 | 0.624 |
| H3 vs. | F | 1 |
|  | M1 | 1 |
|  | M2 | 1 |
|  | Mt | 1 |
|  | H1 | 1 |
|  | H2 | 0.624 |

Supplementary Table S16. Results of linear mixed-effects models (LMMs) evaluating the variation in the Shannon index (alpha diversity) among gut compartments of male adults during the shoot-boring stage. To identify specific compartmental differences, post-hoc pairwise comparisons were conducted, and the family-wise error rate was controlled by applying the Bonferroni adjustment to all derived *P*-values

| **Shannon** | ***F* = 3.13** | ***p*_value = 0.04383278961933715** |
| --- | --- | --- |
|  |  | *p*_value |
| F vs. | M1 | 1 |
|  | M2 | 1 |
|  | Mt | 1 |
|  | H1 | 1 |
|  | H2 | 0.051 |
|  | H3 | 1 |
| M1 vs. | F | 1 |
|  | M2 | 1 |
|  | Mt | 1 |
|  | H1 | 1 |
|  | H2 | 0.523 |
|  | H3 | 1 |
| M2 vs. | F | 1 |
|  | M1 | 1 |
|  | Mt | 1 |
|  | H1 | 1 |
|  | H2 | 0.12 |
|  | H3 | 1 |
| Mt vs. | F | 1 |
|  | M1 | 1 |
|  | M2 | 1 |
|  | H1 | 1 |
|  | H2 | 0.131 |
|  | H3 | 1 |
| H1 vs. | F | 1 |
|  | M1 | 1 |
|  | M2 | 1 |
|  | Mt | 1 |
|  | H2 | 0.506 |
|  | H3 | 1 |
| H2 vs. | F | 0.051 |
|  | M1 | 0.523 |
|  | M2 | 0.12 |
|  | Mt | 0.131 |
|  | H1 | 0.506 |
|  | H3 | 0.283 |
| H3 vs. | F | 1 |
|  | M1 | 1 |
|  | M2 | 1 |
|  | Mt | 1 |
|  | H1 | 1 |
|  | H2 | 0.283 |

Supplementary Table S17. Results of linear mixed-effects models (LMMs) evaluating the variation in the Simpson index (alpha diversity) among gut compartments of male adults during the shoot-boring stage. To identify specific compartmental differences, post-hoc pairwise comparisons were conducted, and the family-wise error rate was controlled by applying the Bonferroni adjustment to all derived *P*-values

| **Simpson** | ***F* = 1.577** | ***p*_value = 0.23613263748482113** |
| --- | --- | --- |
|  |  | *p*_value |
| F vs. | M1 | 1 |
|  | M2 | 1 |
|  | Mt | 1 |
|  | H1 | 1 |
|  | H2 | 0.613 |
|  | H3 | 1 |
| M1 vs. | F | 1 |
|  | M2 | 1 |
|  | Mt | 1 |
|  | H1 | 1 |
|  | H2 | 1 |
|  | H3 | 1 |
| M2 vs. | F | 1 |
|  | M1 | 1 |
|  | Mt | 1 |
|  | H1 | 1 |
|  | H2 | 1 |
|  | H3 | 1 |
| Mt vs. | F | 1 |
|  | M1 | 1 |
|  | M2 | 1 |
|  | H1 | 1 |
|  | H2 | 0.872 |
|  | H3 | 1 |
| H1 vs. | F | 1 |
|  | M1 | 1 |
|  | M2 | 1 |
|  | Mt | 1 |
|  | H2 | 1 |
|  | H3 | 1 |
| H2 vs. | F | 0.613 |
|  | M1 | 1 |
|  | M2 | 1 |
|  | Mt | 0.872 |
|  | H1 | 1 |
|  | H3 | 1 |
| H3 vs. | F | 1 |
|  | M1 | 1 |
|  | M2 | 1 |
|  | Mt | 1 |
|  | H1 | 1 |
|  | H2 | 1 |

Supplementary Table S18. Results of linear mixed-effects models (LMMs) evaluating the variation in the Pielou index (alpha diversity) among gut compartments of male adults during the shoot-boring stage. To identify specific compartmental differences, post-hoc pairwise comparisons were conducted, and the family-wise error rate was controlled by applying the Bonferroni adjustment to all derived *P*-values

| **Pielou** | ***F* = 1.806** | ***p*_value = 0.18049452682403622** |
| --- | --- | --- |
|  |  | *p*_value |
| F vs. | M1 | 1 |
|  | M2 | 1 |
|  | Mt | 1 |
|  | H1 | 1 |
|  | H2 | 0.986 |
|  | H3 | 1 |
| M1 vs. | F | 1 |
|  | M2 | 1 |
|  | Mt | 1 |
|  | H1 | 1 |
|  | H2 | 1 |
|  | H3 | 1 |
| M2 vs. | F | 1 |
|  | M1 | 1 |
|  | Mt | 1 |
|  | H1 | 1 |
|  | H2 | 0.673 |
|  | H3 | 1 |
| Mt vs. | F | 1 |
|  | M1 | 1 |
|  | M2 | 1 |
|  | H1 | 1 |
|  | H2 | 0.423 |
|  | H3 | 1 |
| H1 vs. | F | 1 |
|  | M1 | 1 |
|  | M2 | 1 |
|  | Mt | 1 |
|  | H2 | 1 |
|  | H3 | 1 |
| H2 vs. | F | 0.986 |
|  | M1 | 1 |
|  | M2 | 0.673 |
|  | Mt | 0.423 |
|  | H1 | 1 |
|  | H3 | 1 |
| H3 vs. | F | 1 |
|  | M1 | 1 |
|  | M2 | 1 |
|  | Mt | 1 |
|  | H1 | 1 |
|  | H2 | 1 |

Supplementary Table S19. Results of linear mixed-effects models (LMMs) evaluating the variation in the Sobs index (alpha diversity) among gut compartments of female adults during the shoot-boring stage. To identify specific compartmental differences, post-hoc pairwise comparisons were conducted, and the family-wise error rate was controlled by applying the Bonferroni adjustment to all derived *P*-values

| **Sobs** | ***F* = 7.526** | ***p*_value = 0.001614747995599835** |
| --- | --- | --- |
|  |  | *p*_value |
| F vs. | M1 | 1 |
|  | M2 | 1 |
|  | Mt | 1 |
|  | H1 | 0.003 |
|  | H2 | 1 |
|  | H3 | 1 |
| M1 vs. | F | 1 |
|  | M2 | 1 |
|  | Mt | 1 |
|  | H1 | 0.006 |
|  | H2 | 1 |
|  | H3 | 1 |
| M2 vs. | F | 1 |
|  | M1 | 1 |
|  | Mt | 1 |
|  | H1 | 0.003 |
|  | H2 | 1 |
|  | H3 | 1 |
| Mt vs. | F | 1 |
|  | M1 | 1 |
|  | M2 | 1 |
|  | H1 | 0.005 |
|  | H2 | 1 |
|  | H3 | 1 |
| H1 vs. | F | 0.003 |
|  | M1 | 0.006 |
|  | M2 | 0.003 |
|  | Mt | 0.005 |
|  | H2 | 0.01 |
|  | H3 | 0.011 |
| H2 vs. | F | 1 |
|  | M1 | 1 |
|  | M2 | 1 |
|  | Mt | 1 |
|  | H1 | 0.01 |
|  | H3 | 1 |
| H3 vs. | F | 1 |
|  | M1 | 1 |
|  | M2 | 1 |
|  | Mt | 1 |
|  | H1 | 0.011 |
|  | H2 | 1 |

Supplementary Table S20. Results of linear mixed-effects models (LMMs) evaluating the variation in the Ace index (alpha diversity) among gut compartments of female adults during the shoot-boring stage. To identify specific compartmental differences, post-hoc pairwise comparisons were conducted, and the family-wise error rate was controlled by applying the Bonferroni adjustment to all derived *P*-values

| **Ace** | ***F* = 3.229** | ***p*_value = 0.039818600140673906** |
| --- | --- | --- |
|  |  | *p*_value |
| F vs. | M1 | 1 |
|  | M2 | 1 |
|  | Mt | 1 |
|  | H1 | 0.169 |
|  | H2 | 1 |
|  | H3 | 1 |
| M1 vs. | F | 1 |
|  | M2 | 1 |
|  | Mt | 1 |
|  | H1 | 0.223 |
|  | H2 | 1 |
|  | H3 | 1 |
| M2 vs. | F | 1 |
|  | M1 | 1 |
|  | Mt | 1 |
|  | H1 | 0.261 |
|  | H2 | 1 |
|  | H3 | 1 |
| Mt vs. | F | 1 |
|  | M1 | 1 |
|  | M2 | 1 |
|  | H1 | 0.191 |
|  | H2 | 1 |
|  | H3 | 1 |
| H1 vs. | F | 0.169 |
|  | M1 | 0.223 |
|  | M2 | 0.261 |
|  | Mt | 0.191 |
|  | H2 | 1 |
|  | H3 | 0.267 |
| H2 vs. | F | 1 |
|  | M1 | 1 |
|  | M2 | 1 |
|  | Mt | 1 |
|  | H1 | 1 |
|  | H3 | 1 |
| H3 vs. | F | 1 |
|  | M1 | 1 |
|  | M2 | 1 |
|  | Mt | 1 |
|  | H1 | 0.267 |
|  | H2 | 1 |

Supplementary Table S21. Results of linear mixed-effects models (LMMs) evaluating the variation in the Chao index (alpha diversity) among gut compartments of female adults during the shoot-boring stage. To identify specific compartmental differences, post-hoc pairwise comparisons were conducted, and the family-wise error rate was controlled by applying the Bonferroni adjustment to all derived *P*-values

| **Chao** | ***F* = 4.074** | ***p*_value = 0.018436165143720425** |
| --- | --- | --- |
|  |  | *p*_value |
| F vs. | M1 | 1 |
|  | M2 | 1 |
|  | Mt | 1 |
|  | H1 | 0.043 |
|  | H2 | 1 |
|  | H3 | 1 |
| M1 vs. | F | 1 |
|  | M2 | 1 |
|  | Mt | 1 |
|  | H1 | 0.055 |
|  | H2 | 1 |
|  | H3 | 1 |
| M2 vs. | F | 1 |
|  | M1 | 1 |
|  | Mt | 1 |
|  | H1 | 0.057 |
|  | H2 | 1 |
|  | H3 | 1 |
| Mt vs. | F | 1 |
|  | M1 | 1 |
|  | M2 | 1 |
|  | H1 | 0.071 |
|  | H2 | 1 |
|  | H3 | 1 |
| H1 vs. | F | 0.043 |
|  | M1 | 0.055 |
|  | M2 | 0.057 |
|  | Mt | 0.071 |
|  | H2 | 0.957 |
|  | H3 | 0.102 |
| H2 vs. | F | 1 |
|  | M1 | 1 |
|  | M2 | 1 |
|  | Mt | 1 |
|  | H1 | 0.957 |
|  | H3 | 1 |
| H3 vs. | F | 1 |
|  | M1 | 1 |
|  | M2 | 1 |
|  | Mt | 1 |
|  | H1 | 0.102 |
|  | H2 | 1 |

Supplementary Table S22. Results of linear mixed-effects models (LMMs) evaluating the variation in the Shannon index (alpha diversity) among gut compartments of female adults during the shoot-boring stage. To identify specific compartmental differences, post-hoc pairwise comparisons were conducted, and the family-wise error rate was controlled by applying the Bonferroni adjustment to all derived *P*-values

| **Shannon** | ***F* = 2.419** | ***p*_value = 0.09090409529435181** |
| --- | --- | --- |
|  |  | *p*_value |
| F vs. | M1 | 1 |
|  | M2 | 1 |
|  | Mt | 1 |
|  | H1 | 1 |
|  | H2 | 1 |
|  | H3 | 1 |
| M1 vs. | F | 1 |
|  | M2 | 1 |
|  | Mt | 1 |
|  | H1 | 0.491 |
|  | H2 | 1 |
|  | H3 | 1 |
| M2 vs. | F | 1 |
|  | M1 | 1 |
|  | Mt | 1 |
|  | H1 | 0.118 |
|  | H2 | 1 |
|  | H3 | 1 |
| Mt vs. | F | 1 |
|  | M1 | 1 |
|  | M2 | 1 |
|  | H1 | 0.232 |
|  | H2 | 1 |
|  | H3 | 1 |
| H1 vs. | F | 1 |
|  | M1 | 0.491 |
|  | M2 | 0.118 |
|  | Mt | 0.232 |
|  | H2 | 0.788 |
|  | H3 | 0.678 |
| H2 vs. | F | 1 |
|  | M1 | 1 |
|  | M2 | 1 |
|  | Mt | 1 |
|  | H1 | 0.788 |
|  | H3 | 1 |
| H3 vs. | F | 1 |
|  | M1 | 1 |
|  | M2 | 1 |
|  | Mt | 1 |
|  | H1 | 0.678 |
|  | H2 | 1 |

Supplementary Table S23. Results of linear mixed-effects models (LMMs) evaluating the variation in the Simpson index (alpha diversity) among gut compartments of female adults during the shoot-boring stage. To identify specific compartmental differences, post-hoc pairwise comparisons were conducted, and the family-wise error rate was controlled by applying the Bonferroni adjustment to all derived *P*-values

| **Simpson** | ***F* = 1.327** | ***p*_value = 0.3178542521893916** |
| --- | --- | --- |
|  |  | *p*_value |
| F vs. | M1 | 1 |
|  | M2 | 1 |
|  | Mt | 1 |
|  | H1 | 1 |
|  | H2 | 1 |
|  | H3 | 1 |
| M1 vs. | F | 1 |
|  | M2 | 1 |
|  | Mt | 1 |
|  | H1 | 1 |
|  | H2 | 1 |
|  | H3 | 1 |
| M2 vs. | F | 1 |
|  | M1 | 1 |
|  | Mt | 1 |
|  | H1 | 0.896 |
|  | H2 | 1 |
|  | H3 | 1 |
| Mt vs. | F | 1 |
|  | M1 | 1 |
|  | M2 | 1 |
|  | H1 | 1 |
|  | H2 | 1 |
|  | H3 | 1 |
| H1 vs. | F | 1 |
|  | M1 | 1 |
|  | M2 | 0.896 |
|  | Mt | 1 |
|  | H2 | 1 |
|  | H3 | 1 |
| H2 vs. | F | 1 |
|  | M1 | 1 |
|  | M2 | 1 |
|  | Mt | 1 |
|  | H1 | 1 |
|  | H3 | 1 |
| H3 vs. | F | 1 |
|  | M1 | 1 |
|  | M2 | 1 |
|  | Mt | 1 |
|  | H1 | 1 |
|  | H2 | 1 |

Supplementary Table S24. Results of linear mixed-effects models (LMMs) evaluating the variation in the Pielou index (alpha diversity) among gut compartments of female adults during the shoot-boring stage. To identify specific compartmental differences, post-hoc pairwise comparisons were conducted, and the family-wise error rate was controlled by applying the Bonferroni adjustment to all derived *P*-values

| **Pielou** | ***F* = 2.138** | ***p*_value = 0.1237738229548047** |
| --- | --- | --- |
|  |  | *p*_value |
| F vs. | M1 | 1 |
|  | M2 | 0.249 |
|  | Mt | 0.344 |
|  | H1 | 1 |
|  | H2 | 1 |
|  | H3 | 1 |
| M1 vs. | F | 1 |
|  | M2 | 1 |
|  | Mt | 1 |
|  | H1 | 1 |
|  | H2 | 1 |
|  | H3 | 1 |
| M2 vs. | F | 0.249 |
|  | M1 | 1 |
|  | Mt | 1 |
|  | H1 | 1 |
|  | H2 | 1 |
|  | H3 | 1 |
| Mt vs. | F | 0.344 |
|  | M1 | 1 |
|  | M2 | 1 |
|  | H1 | 1 |
|  | H2 | 1 |
|  | H3 | 1 |
| H1 vs. | F | 1 |
|  | M1 | 1 |
|  | M2 | 1 |
|  | Mt | 1 |
|  | H2 | 1 |
|  | H3 | 1 |
| H2 vs. | F | 1 |
|  | M1 | 1 |
|  | M2 | 1 |
|  | Mt | 1 |
|  | H1 | 1 |
|  | H3 | 1 |
| H3 vs. | F | 1 |
|  | M1 | 1 |
|  | M2 | 1 |
|  | Mt | 1 |
|  | H1 | 1 |
|  | H2 | 1 |

Supplementary Table S25. Results of linear mixed‑effects models testing the sex‑by‑compartment interaction on the Sobs index across seven gut segments during the shoot‑boring stage. Post‑hoc pairwise comparisons, adjusted with Bonferroni correction, are also included to dissect the interaction for each sex‑specific comparison.

| **Sobs** | ***F* = 2.919** | ***p*_value = 0.0277848990448041** |
| --- | --- | --- |
| **Female vs. Male** | ***F*** | ***p*_value** |
| F | 0.016 | 0.9 |
| M1 | 0.197 | 0.662 |
| M2 | 0.681 | 0.419 |
| Mt | 1.119 | 0.303 |
| H1 | 0.28 | 0.603 |
| H2 | 16.497 | 6.286755571898795E-4 |
| H3 | 0.646 | 0.431 |

Supplementary Table S26. Results of linear mixed‑effects models testing the sex‑by‑compartment interaction on the Ace index across seven gut segments during the shoot‑boring stage. Post‑hoc pairwise comparisons, adjusted with Bonferroni correction, are also included to dissect the interaction for each sex‑specific comparison.

| **Ace** | ***F* = 0.555** | ***p*_value = 0.7619115760175799** |
| --- | --- | --- |
| **Female vs. Male** | ***F*** | ***p*_value** |
| F | 0.004 | 0.949 |
| M1 | 0.029 | 0.866 |
| M2 | 3.785 | 0.062 |
| Mt | 0.912 | 0.348 |
| H1 | 0.805 | 0.377 |
| H2 | 3.324 | 0.079 |
| H3 | 0.26 | 0.614 |

Supplementary Table S27. Results of linear mixed‑effects models testing the sex‑by‑compartment interaction on the Chao index across seven gut segments during the shoot‑boring stage. Post‑hoc pairwise comparisons, adjusted with Bonferroni correction, are also included to dissect the interaction for each sex‑specific comparison

| **Chao** | ***F* = 1.268** | ***p*_value = 0.3089508352103493** |
| --- | --- | --- |
| **Female vs. Male** | ***F*** | ***p*_value** |
| F | 0.005 | 0.942 |
| M1 | 0.067 | 0.797 |
| M2 | 0.554 | 0.464 |
| Mt | 0.907 | 0.35 |
| H1 | 0.005 | 0.946 |
| H2 | 9.404 | 0.005 |
| H3 | 0.454 | 0.507 |

Supplementary Table S28. Results of linear mixed‑effects models testing the sex‑by‑compartment interaction on the Shannon index across seven gut segments during the shoot‑boring stage. Post‑hoc pairwise comparisons, adjusted with Bonferroni correction, are also included to dissect the interaction for each sex‑specific comparison

| **Shannon** | ***F* = 2.703** | ***p*_value = 0.037749288481764814** |
| --- | --- | --- |
| **Female vs. Male** | ***F*** | ***p*_value** |
| F | 0.01 | 0.923 |
| M1 | 1.566 | 0.243 |
| M2 | 1.11 | 0.32 |
| Mt | 0.82 | 0.389 |
| H1 | 0.006 | 0.942 |
| H2 | 9.089 | 0.015 |
| H3 | 0.843 | 0.383 |

Supplementary Table S29. Results of linear mixed‑effects models testing the sex‑by‑compartment interaction on the Simpson index across seven gut segments during the shoot‑boring stage. Post‑hoc pairwise comparisons, adjusted with Bonferroni correction, are also included to dissect the interaction for each sex‑specific comparison

| **Simpson** | ***F* = 0.912** | ***p*_value = 0.5034307915172033** |
| --- | --- | --- |
| **Female vs. Male** | ***F*** | ***p*_value** |
| F | 0.033 | 0.86 |
| M1 | 1.78 | 0.215 |
| M2 | 1.32 | 0.28 |
| Mt | 0.937 | 0.358 |
| H1 | 0.104 | 0.755 |
| H2 | 2.572 | 0.143 |
| H3 | 0.871 | 0.375 |

Supplementary Table S30. Results of linear mixed‑effects models testing the sex‑by‑compartment interaction on the Pielou index across seven gut segments during the shoot‑boring stage. Post‑hoc pairwise comparisons, adjusted with Bonferroni correction, are also included to dissect the interaction for each sex‑specific comparison

| **Pielou** | ***F* = 1.804** | ***p*_value = 0.14072923819872032** |
| --- | --- | --- |
| **Female vs. Male** | ***F*** | ***p*_value** |
| F | 0.493 | 0.501 |
| M1 | 2.127 | 0.18 |
| M2 | 1.003 | 0.344 |
| Mt | 0.544 | 0.48 |
| H1 | 0.026 | 0.876 |
| H2 | 3.254 | 0.106 |
| H3 | 0.275 | 0.614 |

Supplementary Table S31. Results of linear mixed‑effects models testing the sex‑by‑compartment interaction on the Sobs index across seven gut segments during the trunk‑boring stage. Post‑hoc pairwise comparisons, adjusted with Bonferroni correction, are also included to dissect the interaction for each sex‑specific comparison.

| **Sobs** | ***F* = 7.957** | ***p*_value = 8.550580431765053E-5** |
| --- | --- | --- |
| **Female vs. Male** | ***F*** | ***p*_value** |
| F | 0.683 | 0.415 |
| M1 | 0.481 | 0.494 |
| M2 | 0.344 | 0.562 |
| Mt | 0.257 | 0.616 |
| H1 | 2.647 | 0.115 |
| H2 | 7.255 | 0.012 |
| H3 | 51.084 | 0 |

Supplementary Table S32. Results of linear mixed‑effects models testing the sex‑by‑compartment interaction on the Ace index across seven gut segments during the trunk‑boring stage. Post‑hoc pairwise comparisons, adjusted with Bonferroni correction, are also included to dissect the interaction for each sex‑specific comparison.

| **Ace** | ***F* = 7.039** | **p_value = 2.0681736263150345E-4** |
| --- | --- | --- |
| **Female vs. Male** | ***F*** | **p_value** |
| F | 0.392 | 0.537 |
| M1 | 0.026 | 0.873 |
| M2 | 0.185 | 0.671 |
| Mt | 0.585 | 0.451 |
| H1 | 3.334 | 0.08 |
| H2 | 4.8 | 0.038 |
| H3 | 45.195 | 0 |

Supplementary Table S33. Results of linear mixed‑effects models testing the sex‑by‑compartment interaction on the Chao index across seven gut segments during the trunk‑boring stage. Post‑hoc pairwise comparisons, adjusted with Bonferroni correction, are also included to dissect the interaction for each sex‑specific comparison

| **Chao** | ***F* = 8.069** | ***p*_value = 7.708268871578313E-5** |
| --- | --- | --- |
| **Female vs. Male** | ***F*** | ***p*_value** |
| F | 0.689 | 0.414 |
| M1 | 0.485 | 0.492 |
| M2 | 0.347 | 0.561 |
| Mt | 0.259 | 0.615 |
| H1 | 2.668 | 0.114 |
| H2 | 7.387 | 0.011 |
| H3 | 51.796 | 0 |

Supplementary Table S34. Results of linear mixed‑effects models testing the sex‑by‑compartment interaction on the Shannon index across seven gut segments during the trunk‑boring stage. Post‑hoc pairwise comparisons, adjusted with Bonferroni correction, are also included to dissect the interaction for each sex‑specific comparison

| **Shannon** | ***F* = 1.35** | ***p*_value = 0.2742622512006292** |
| --- | --- | --- |
| **Female vs. Male** | ***F*** | ***p*_value** |
| F | 0.524 | 0.476 |
| M1 | 0.286 | 0.597 |
| M2 | 0.46 | 0.504 |
| Mt | 1.497 | 0.232 |
| H1 | 0.361 | 0.553 |
| H2 | 0.334 | 0.568 |
| H3 | 8.494 | 0.007 |

Supplementary Table S35. Results of linear mixed‑effects models testing the sex‑by‑compartment interaction on the Simpson index across seven gut segments during the trunk‑boring stage. Post‑hoc pairwise comparisons, adjusted with Bonferroni correction, are also included to dissect the interaction for each sex‑specific comparison

| **Simpson** | ***F* = 1.705** | ***p*_value = 0.16317570187629243** |
| --- | --- | --- |
| **Female vs. Male** | ***F*** | ***p*_value** |
| F | 1.096 | 0.305 |
| M1 | 1.007 | 0.325 |
| M2 | 0.288 | 0.596 |
| Mt | 1.973 | 0.172 |
| H1 | 4.319 | 0.048 |
| H2 | 0.447 | 0.51 |
| H3 | 3.871 | 0.06 |

Supplementary Table S36. Results of linear mixed‑effects models testing the sex‑by‑compartment interaction on the Pielou index across seven gut segments during the trunk‑boring stage. Post‑hoc pairwise comparisons, adjusted with Bonferroni correction, are also included to dissect the interaction for each sex‑specific comparison

| **Pielou** | ***F* = 0.927** | ***p*_value = 0.4937188215755145** |
| --- | --- | --- |
| **Female vs. Male** | ***F*** | ***p*_value** |
| F | 0.729 | 0.409 |
| M1 | 1.522 | 0.239 |
| M2 | 0.002 | 0.967 |
| Mt | 0.104 | 0.753 |
| H1 | 1.134 | 0.307 |
| H2 | 0.738 | 0.406 |
| H3 | 0.413 | 0.532 |

Supplementary Table S37. Results of linear mixed‑effects models testing the stage‑by‑compartment interaction on the Sobs index in female adults, across the seven gut segments and between the shoot‑boring and trunk‑boring stages. Post‑hoc pairwise comparisons with Bonferroni adjustment are included to dissect the interaction for each stage‑specific contrast.

| **Sobs** | ***F* = 2.097** | ***p*_value = 0.09132240408500834** |
| --- | --- | --- |
| **Shoot vs. Trunk** | ***F*** | ***p*_value** |
| F | 5.78 | 0.024 |
| M1 | 10.547 | 0.003 |
| M2 | 5.579 | 0.027 |
| Mt | 11.102 | 0.003 |
| H1 | 0.349 | 0.56 |
| H2 | 5.78 | 0.024 |
| H3 | 3.602 | 0.07 |

Supplementary Table S38. Results of linear mixed‑effects models testing the stage‑by‑compartment interaction on the Ace index in female adults, across the seven gut segments and between the shoot‑boring and trunk‑boring stages. Post‑hoc pairwise comparisons with Bonferroni adjustment are included to dissect the interaction for each stage‑specific contrast.

| **Ace** | ***F* = 2.44** | ***p*_value = 0.05520813683765229** |
| --- | --- | --- |
| **Shoot vs. Trunk** | ***F*** | ***p*_value** |
| F | 1.398 | 0.248 |
| M1 | 1.647 | 0.211 |
| M2 | 1.37 | 0.253 |
| Mt | 2.694 | 0.113 |
| H1 | 5.099 | 0.033 |
| H2 | 0.379 | 0.544 |
| H3 | 2.162 | 0.154 |

Supplementary Table S39. Results of linear mixed‑effects models testing the stage‑by‑compartment interaction on the Chao index in female adults, across the seven gut segments and between the shoot‑boring and trunk‑boring stages. Post‑hoc pairwise comparisons with Bonferroni adjustment are included to dissect the interaction for each stage‑specific contrast.

| **Chao** | ***F* = 2.685** | ***p*_value = 0.03875437580863248** |
| --- | --- | --- |
| **Shoot vs. Trunk** | ***F*** | ***p*_value** |
| F | 2.76 | 0.108 |
| M1 | 4.972 | 0.034 |
| M2 | 2.124 | 0.156 |
| Mt | 4.404 | 0.045 |
| H1 | 5.467 | 0.027 |
| H2 | 0 | 0.992 |
| H3 | 1.227 | 0.278 |

Supplementary Table S40. Results of linear mixed‑effects models testing the stage‑by‑compartment interaction on the Shannon index in female adults, across the seven gut segments and between the shoot‑boring and trunk‑boring stages. Post‑hoc pairwise comparisons with Bonferroni adjustment are included to dissect the interaction for each stage‑specific contrast.

| **Shannon** | ***F* = 0.804** | ***p*_value = 0.5768272314026139** |
| --- | --- | --- |
| **Shoot vs. Trunk** | ***F*** | ***p*_value** |
| F | 0.039 | 0.848 |
| M1 | 0.015 | 0.905 |
| M2 | 1.466 | 0.252 |
| Mt | 0.986 | 0.342 |
| H1 | 0.038 | 0.85 |
| H2 | 0.975 | 0.345 |
| H3 | 0.286 | 0.604 |

Supplementary Table S41. Results of linear mixed‑effects models testing the stage‑by‑compartment interaction on the Simpson index in female adults, across the seven gut segments and between the shoot‑boring and trunk‑boring stages. Post‑hoc pairwise comparisons with Bonferroni adjustment are included to dissect the interaction for each stage‑specific contrast.

| **Simpson** | ***F* = 1.832** | ***p*_value = 0.13518840623629555** |
| --- | --- | --- |
| **Shoot vs. Trunk** | ***F*** | ***p*_value** |
| F | 0.008 | 0.931 |
| M1 | 0.015 | 0.905 |
| M2 | 2.245 | 0.165 |
| Mt | 1.387 | 0.266 |
| H1 | 0.657 | 0.436 |
| H2 | 2.298 | 0.161 |
| H3 | 0.367 | 0.558 |

Supplementary Table S42. Results of linear mixed‑effects models testing the stage‑by‑compartment interaction on the Pielou index in female adults, across the seven gut segments and between the shoot‑boring and trunk‑boring stages. Post‑hoc pairwise comparisons with Bonferroni adjustment are included to dissect the interaction for each stage‑specific contrast.

| **Pielou** | ***F* = 2.523** | ***p*_value = 0.048912897697078656** |
| --- | --- | --- |
| **Shoot vs. Trunk** | ***F*** | ***p*_value** |
| F | 2.035 | 0.191 |
| M1 | 0.299 | 0.599 |
| M2 | 0.855 | 0.382 |
| Mt | 0.483 | 0.506 |
| H1 | 0.814 | 0.393 |
| H2 | 1.05 | 0.335 |
| H3 | 0.022 | 0.886 |

Supplementary Table S43. Results of linear mixed‑effects models testing the stage‑by‑compartment interaction on the Sobs index in male adults, across the seven gut segments and between the shoot‑boring and trunk‑boring stages. Post‑hoc pairwise comparisons with Bonferroni adjustment are included to dissect the interaction for each stage‑specific contrast.

| **Sobs** | ***F* = 3.859** | ***p*_value = 0.007763415871694079** |
| --- | --- | --- |
| **Shoot vs. Trunk** | ***F*** | ***p*_value** |
| F | 0.13 | 0.722 |
| M1 | 0.292 | 0.594 |
| M2 | 0.494 | 0.489 |
| Mt | 0.748 | 0.396 |
| H1 | 1.684 | 0.207 |
| H2 | 0.376 | 0.546 |
| H3 | 24.566 | 0 |

Supplementary Table S44. Results of linear mixed‑effects models testing the stage‑by‑compartment interaction on the Ace index in male adults, across the seven gut segments and between the shoot‑boring and trunk‑boring stages. Post‑hoc pairwise comparisons with Bonferroni adjustment are included to dissect the interaction for each stage‑specific contrast.

| **Ace** | ***F* = 1.36** | ***p*_value = 0.2650397712432718** |
| --- | --- | --- |
| **Shoot vs. Trunk** | ***F*** | ***p*_value** |
| F | 0.022 | 0.884 |
| M1 | 0.038 | 0.846 |
| M2 | 2.201 | 0.149 |
| Mt | 0.056 | 0.815 |
| H1 | 1.313 | 0.262 |
| H2 | 2.132 | 0.155 |
| H3 | 3.101 | 0.089 |

Supplementary Table S45. Results of linear mixed‑effects models testing the stage‑by‑compartment interaction on the Chao index in male adults, across the seven gut segments and between the shoot‑boring and trunk‑boring stages. Post‑hoc pairwise comparisons with Bonferroni adjustment are included to dissect the interaction for each stage‑specific contrast.

| **Chao** | ***F* = 2.388** | ***p*_value = 0.0595725875719984** |
| --- | --- | --- |
| **Shoot vs. Trunk** | ***F*** | ***p*_value** |
| F | 0.059 | 0.811 |
| M1 | 0.127 | 0.725 |
| M2 | 0.019 | 0.89 |
| Mt | 0.027 | 0.87 |
| H1 | 0.09 | 0.767 |
| H2 | 3.184 | 0.086 |
| H3 | 9.31 | 0.005 |

Supplementary Table S46. Results of linear mixed‑effects models testing the stage‑by‑compartment interaction on the Shannon index in male adults, across the seven gut segments and between the shoot‑boring and trunk‑boring stages. Post‑hoc pairwise comparisons with Bonferroni adjustment are included to dissect the interaction for each stage‑specific contrast.

| **Shannon** | ***F* = 3.432** | ***p*_value = 0.013696940418094397** |
| --- | --- | --- |
| **Shoot vs. Trunk** | ***F*** | ***p*_value** |
| F | 0.693 | 0.415 |
| M1 | 1.656 | 0.214 |
| M2 | 0.722 | 0.406 |
| Mt | 1.678 | 0.211 |
| H1 | 0.187 | 0.67 |
| H2 | 4.515 | 0.047 |
| H3 | 5.451 | 0.031 |

Supplementary Table S47. Results of linear mixed‑effects models testing the stage‑by‑compartment interaction on the Simpson index in male adults, across the seven gut segments and between the shoot‑boring and trunk‑boring stages. Post‑hoc pairwise comparisons with Bonferroni adjustment are included to dissect the interaction for each stage‑specific contrast.

| **Simpson** | ***F* = 2.097** | ***p*_value = 0.09130490979709448** |
| --- | --- | --- |
| **Shoot vs. Trunk** | ***F*** | ***p*_value** |
| F | 1.003 | 0.326 |
| M1 | 1.679 | 0.207 |
| M2 | 1.565 | 0.222 |
| Mt | 3.815 | 0.062 |
| H1 | 0.137 | 0.714 |
| H2 | 0.617 | 0.44 |
| H3 | 2.738 | 0.11 |

Supplementary Table S48. Results of linear mixed‑effects models testing the stage‑by‑compartment interaction on the Pielou index in male adults, across the seven gut segments and between the shoot‑boring and trunk‑boring stages. Post‑hoc pairwise comparisons with Bonferroni adjustment are included to dissect the interaction for each stage‑specific contrast.

| **Pielou** | ***F* = 1.173** | ***p*_value = 0.35341394280672217** |
| --- | --- | --- |
| **Shoot vs. Trunk** | ***F*** | ***p*_value** |
| F | 0.035 | 0.855 |
| M1 | 2.556 | 0.131 |
| M2 | 0.002 | 0.967 |
| Mt | 0.084 | 0.776 |
| H1 | 0.192 | 0.668 |
| H2 | 3.906 | 0.067 |
| H3 | 0.088 | 0.771 |

Supplementary Table S49. Per‑sample read processing metrics in trunk‑boring stage: raw reads, quality‑filtered reads, non‑chimeric reads, and final ASV counts.

| **Sample** | **Raw_Reads** | **Filtered_Reads** | **NonChimeric_Reads** | **ASVs_num** |
| --- | --- | --- | --- | --- |
| BF_F_1 | 68633 | 62082 | 49513 | 33 |
| BF_F_2 | 68075 | 61097 | 56578 | 42 |
| BF_F_3 | 63926 | 57607 | 53070 | 53 |
| BF_M1_1 | 67897 | 60803 | 50224 | 36 |
| BF_M1_2 | 71896 | 64742 | 59676 | 44 |
| BF_M1_3 | 82195 | 73910 | 70007 | 54 |
| BF_M2_1 | 60640 | 54672 | 39416 | 30 |
| BF_M2_2 | 72911 | 65327 | 60996 | 45 |
| BF_M2_3 | 67151 | 60252 | 55689 | 78 |
| BF_Mt_1 | 62396 | 56381 | 49499 | 56 |
| BF_Mt_2 | 73561 | 65814 | 61846 | 50 |
| BF_Mt_3 | 76366 | 69391 | 63667 | 57 |
| BF_H1_1 | 60454 | 55015 | 49493 | 75 |
| BF_H1_2 | 63523 | 56895 | 52228 | 29 |
| BF_H1_3 | 66678 | 57146 | 52307 | 81 |
| BF_H2_1 | 62535 | 56170 | 45010 | 61 |
| BF_H2_2 | 69402 | 62612 | 59163 | 38 |
| BF_H2_3 | 62727 | 55286 | 50616 | 129 |
| BF_H3_1 | 58045 | 52685 | 45764 | 55 |
| BF_H3_2 | 72271 | 65387 | 61822 | 38 |
| BF_H3_3 | 76192 | 68655 | 63107 | 76 |
| BM_F_1 | 67670 | 60890 | 52531 | 28 |
| BM_F_2 | 71120 | 63384 | 57715 | 26 |
| BM_F_3 | 73641 | 66380 | 60199 | 34 |
| BM_M1_1 | 59577 | 53335 | 45411 | 39 |
| BM_M1_2 | 61728 | 55602 | 50396 | 47 |
| BM_M1_3 | 73854 | 66217 | 59002 | 46 |
| BM_M2_1 | 80771 | 72989 | 61488 | 33 |
| BM_M2_2 | 53227 | 47194 | 42963 | 83 |
| BM_M2_3 | 57016 | 51365 | 46761 | 76 |
| BM_Mt_1 | 46724 | 41764 | 37918 | 61 |
| BM_Mt_2 | 69860 | 62448 | 57161 | 77 |
| BM_Mt_3 | 71247 | 64360 | 54836 | 84 |
| BM_H1_1 | 57449 | 51622 | 47057 | 104 |
| BM_H1_2 | 69734 | 63104 | 58081 | 104 |
| BM_H1_3 | 61022 | 55795 | 50862 | 59 |
| BM_H2_1 | 59866 | 54483 | 47665 | 152 |
| BM_H2_2 | 62464 | 56007 | 51715 | 130 |
| BM_H2_3 | 73214 | 65897 | 57787 | 71 |
| BM_H3_1 | 77317 | 69425 | 63486 | 237 |
| BM_H3_2 | 75494 | 67763 | 63620 | 179 |
| BM_H3_3 | 74933 | 67636 | 59603 | 164 |
| Summary | 2825402 | 2539589 | 2275948 | 2994 |
| Average | 67271.47619 | 60466.40476 | 54189.2381 | 71.28571429 |

Supplementary Table S50. Per‑sample read processing metrics in shoot‑boring stage: raw reads, quality‑filtered reads, non‑chimeric reads, and final ASV counts.

| **Sample** | **Raw_Reads** | **Filtered_Reads** | **NonChimeric_Reads** | **ASVs_num** |
| --- | --- | --- | --- | --- |
| BF_F_1 | 74773 | 67764 | 54431 | 123 |
| BF_F_2 | 73966 | 66509 | 50610 | 49 |
| BF_F_3 | 75251 | 67827 | 56163 | 109 |
| BF_M1_1 | 81858 | 73888 | 61331 | 108 |
| BF_M1_2 | 65335 | 59050 | 46751 | 25 |
| BF_M1_3 | 71960 | 65206 | 55359 | 110 |
| BF_M2_1 | 77994 | 70708 | 58449 | 46 |
| BF_M2_2 | 64951 | 58319 | 47509 | 42 |
| BF_M2_3 | 65304 | 58750 | 48279 | 85 |
| BF_Mt_1 | 70818 | 64775 | 53628 | 34 |
| BF_Mt_2 | 66810 | 60628 | 49321 | 41 |
| BF_Mt_3 | 76379 | 69162 | 56168 | 19 |
| BF_H1_1 | 68395 | 61782 | 51376 | 136 |
| BF_H1_2 | 55880 | 49881 | 40638 | 59 |
| BF_H1_3 | 59222 | 53724 | 44594 | 123 |
| BF_H2_1 | 83203 | 74741 | 62091 | 25 |
| BF_H2_2 | 71915 | 64740 | 51401 | 54 |
| BF_H2_3 | 303802 | 272638 | 241397 | 115 |
| BF_H3_1 | 66120 | 59709 | 50137 | 45 |
| BF_H3_2 | 72729 | 65732 | 52988 | 63 |
| BF_H3_3 | 70187 | 64289 | 52813 | 100 |
| BM_F_1 | 73852 | 66655 | 55708 | 132 |
| BM_F_2 | 72982 | 64928 | 54762 | 83 |
| BM_F_3 | 83995 | 75647 | 61613 | 120 |
| BM_M1_1 | 75878 | 68607 | 56378 | 126 |
| BM_M1_2 | 70737 | 63895 | 52670 | 99 |
| BM_M1_3 | 73098 | 65545 | 53346 | 126 |
| BM_M2_1 | 68969 | 62412 | 52069 | 116 |
| BM_M2_2 | 61005 | 54731 | 46241 | 69 |
| BM_M2_3 | 73018 | 65897 | 54992 | 105 |
| BM_Mt_1 | 69371 | 62778 | 52371 | 137 |
| BM_Mt_2 | 293890 | 267869 | 236552 | 141 |
| BM_Mt_3 | 68645 | 62162 | 50862 | 75 |
| BM_H1_1 | 67502 | 55824 | 47038 | 129 |
| BM_H1_2 | 310932 | 281151 | 245722 | 220 |
| BM_H1_3 | 71028 | 64321 | 52118 | 82 |
| BM_H2_1 | 67039 | 60472 | 49753 | 371 |
| BM_H2_2 | 60784 | 55024 | 45909 | 110 |
| BM_H2_3 | 66923 | 61065 | 49912 | 126 |
| BM_H3_1 | 67327 | 61425 | 50506 | 170 |
| BM_H3_2 | 68973 | 62575 | 51394 | 98 |
| BM_H3_3 | 63360 | 57330 | 46702 | 115 |
| Summary‌ | 3646160 | 3290135 | 2752052 | 4261 |
| Average | 86813.33333 | 78336.54762 | 65525.04762 | 101.452381 |

Supplementary Table S51. Principal coordinates analysis (PCoA) based on Bray-Curtis dissimilarity and tested by PERMANOVA (adonis2) for seven gut compartments of trunk‑boring stage males.

| **R^2^=0.5870** | ***P*=0.014** |
| --- | --- |
| **group** | ***P*** |
| BM_H3 vs. BM_H2 | >0.05 |
| BM_H3 vs. BM_H1 | >0.05 |
| BM_H3 vs. BM_M1 | <0.05 |
| BM_H3 vs. BM_M2 | >0.05 |
| BM_H3 vs. BM_Mt | >0.05 |
| BM_H3 vs. BM_F | <0.05 |
| BM_H2 vs. BM_H1 | >0.05 |
| BM_H2 vs. BM_M1 | <0.05 |
| BM_H2 vs. BM_M2 | >0.05 |
| BM_H2 vs. BM_Mt | >0.05 |
| BM_H2 vs. BM_F | <0.05 |
| BM_H1 vs. BM_M1 | <0.05 |
| BM_H1 vs. BM_M2 | >0.05 |
| BM_H1 vs. BM_Mt | >0.05 |
| BM_H1 vs. BM_F | <0.05 |
| BM_M1 vs. BM_M2 | >0.05 |
| BM_M1 vs. BM_Mt | <0.05 |
| BM_M1 vs. BM_F | >0.05 |
| BM_M2 vs. BM_Mt | >0.05 |
| BM_M2 vs. BM_F | >0.05 |
| BM_Mt vs. BM_F | <0.05 |
